# Watchdog 2.0: New developments for reusability, reproducibility, and workflow execution

**DOI:** 10.1093/gigascience/giaa068

**Published:** 2020-06-17

**Authors:** Michael Kluge, Marie-Sophie Friedl, Amrei L Menzel, Caroline C Friedel

**Affiliations:** Institut für Informatik, Ludwig-Maximilians-Universität München, Amalienstr. 17, Munich 80333, Germany

**Keywords:** workflow management system, bioinformatics, automated biological data analysis, next-generation sequencing, reusability, reproducibility, open science tools

## Abstract

**Background:**

Advances in high-throughput methods have brought new challenges for biological data analysis, often requiring many interdependent steps applied to a large number of samples. To address this challenge, workflow management systems, such as Watchdog, have been developed to support scientists in the (semi-)automated execution of large analysis workflows.

**Implementation:**

Here, we present Watchdog 2.0, which implements new developments for module creation, reusability, and documentation and for reproducibility of analyses and workflow execution. Developments include a graphical user interface for semi-automatic module creation from software help pages, sharing repositories for modules and workflows, and a standardized module documentation format. The latter allows generation of a customized reference book of public and user-specific modules. Furthermore, extensive logging of workflow execution, module and software versions, and explicit support for package managers and container virtualization now ensures reproducibility of results. A step-by-step analysis protocol generated from the log file may, e.g., serve as a draft of a manuscript methods section. Finally, 2 new execution modes were implemented. One allows resuming workflow execution after interruption or modification without rerunning successfully executed tasks not affected by changes. The second one allows detaching and reattaching to workflow execution on a local computer while tasks continue running on computer clusters.

**Conclusions:**

Watchdog 2.0 provides several new developments that we believe to be of benefit for large-scale bioinformatics analysis and that are not completely covered by other competing workflow management systems. The software itself, module and workflow repositories, and comprehensive documentation are freely available at https://www.bio.ifi.lmu.de/watchdog.

## Background

As a result of improvements in sequencing technologies, sequencing costs have decreased massively in recent years [1]. While the first human genome sequence cost ∼$2.7 billion and took 13 years to complete [2], companies now offer genome sequencing to private customers using state-of-the-art next-generation sequencing (NGS) technologies for <$1,000. In addition, other cellular properties can now be measured at large scale using NGS. This includes, e.g., the expression of genes (RNA sequencing [RNA-seq]) [3], protein binding to DNA (chromatin immunoprecipitation sequencing [ChIP-seq]) [4], open chromatin regions (assay for transposase-accessible chromatin using sequencing [ATAC-seq]) [5], and many more.

As a consequence, data analysis has become more complex with new challenges for bioinformatics, often requiring multiple interdependent steps and integration of numerous replicates and several types of high-throughput data. Because manual execution of all required analysis steps is cumbersome, time-consuming, and laborious to repeat, several tools have been developed for performing large-scale bioinformatics analyses. One group of tools consists of static analysis pipelines specifically designed for 1 application, e.g., transcriptome analysis [6,7]. While these pipelines have the advantage that a particular analysis can be repeated without great effort, components of these analysis pipelines are often not easily reusable for other related applications. As an alternative, workflow management systems (WMSs) have been developed that support creation of such analysis pipelines (denoted as workflows in this context) from reusable components and allow (semi-)automated execution of these workflows. Popular WMSs are Galaxy [8], KNIME [9], Snakemake [10], and Nextflow [11] and differ in the implemented set of features, target audience, the required training period, usage fees, and more (for more details see the comparison in the first article on Watchdog [12] and at the end of this article).

Previously, we presented the WMS Watchdog for the distributed analysis of large-scale experimental data originating, e.g., from NGS experiments [12]. The core features of Watchdog include straightforward processing of replicate data, support for and flexible combination of distributed computing or remote executors, customizable error detection, user notification on execution errors, and manual user intervention. In Watchdog, reusable components are encapsulated within so-called modules, which are defined by an XSD file specifying the program to execute, input parameters, and return values of the module. In addition, modules can contain scripts or compiled binaries that are invoked in the module. There are no restrictions on included software or on the programming language used in additional scripts. Modules may also deploy required software internally using Conda [13], Docker [14], or similar tools.

A Watchdog workflow is defined in an XML format and consists of a sequence of tasks and dependencies between tasks. Each task uses 1 module and the same task can be automatically run on multiple samples or with multiple parameter combinations using so-called process blocks. This creates several subtasks, 1 for each sample or parameter combination. A workflow can either be created manually using any XML editor or the Watchdog graphical user interface (GUI) for workflow construction. While XML may be more complex than, e.g., YAML or JSON, it is widely used and numerous XML editors are available, e.g., plugins for Eclipse [15]. Furthermore, using the GUI requires neither understanding of XML nor programming skills and thus allows easy construction of workflows from a pre-defined set of modules. In this case, the only Watchdog syntax that has to be learned is how to reference variables.

Workflows can be executed using the Watchdog scheduler via a command-line interface or the GUI, which are both implemented in Java and thus platform-independent. The Watchdog scheduler continuously monitors the execution status of tasks and schedules new tasks or subtasks for execution if all tasks that they depend on finished successfully. The execution status of tasks is reported to the user via standard output, a web interface that allows manual intervention and (optionally) email.

In the workflow, different executors can be specified for different tasks. Currently, 3 types of executors are supported (local host, remote host via SSH, or computer clusters using SGE or SLURM). Thus, resource-intensive or long-running tasks can, e.g., be submitted to a computer cluster while less demanding tasks may be executed on the local host. Furthermore, Watchdog provides a plugin system that allows users with programming skills to add new executor types, e.g., for cloud computing, without having to change the original Watchdog code (for details see [12]).

In this article, we present Watchdog 2.0, a new and improved version of Watchdog with several new developments for module creation and documentation, reusability of modules and workflows, and reproducibility of analysis results, as well as workflow execution.

## Implementation

### Overview

**Figure 1:**
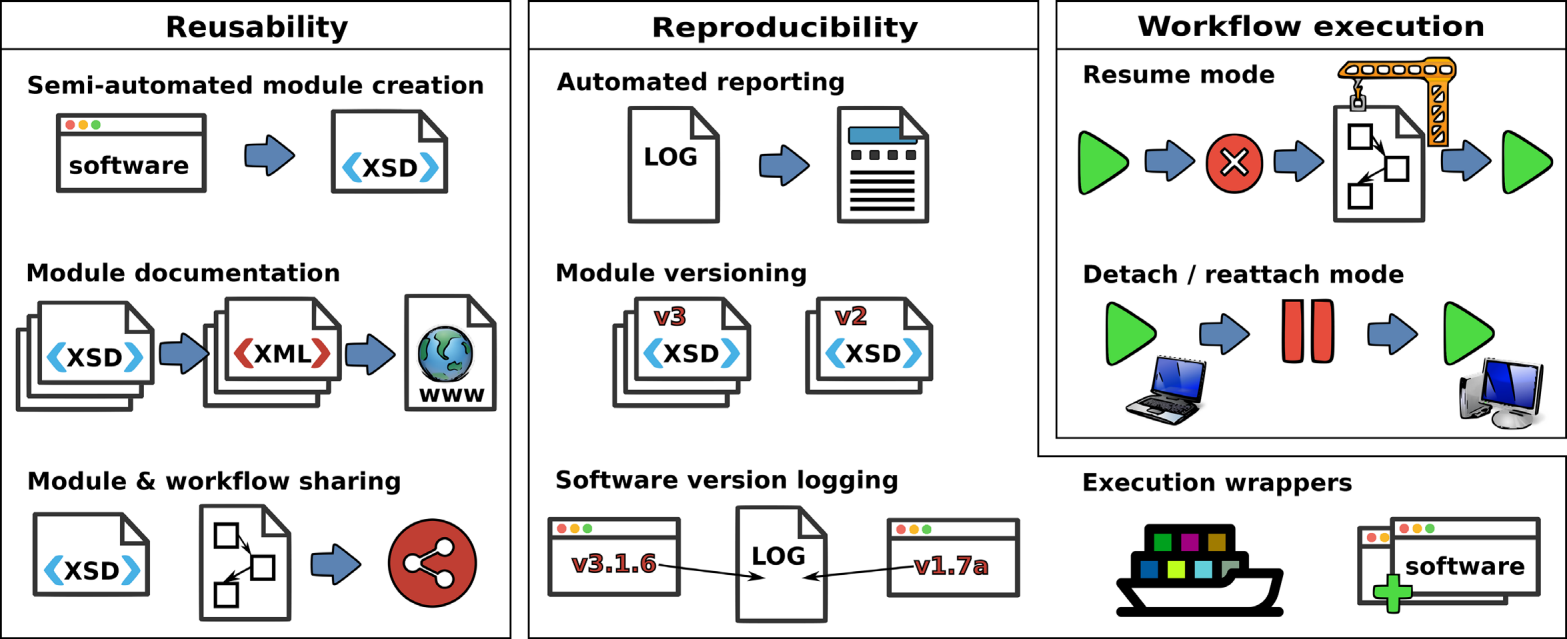
Overview on new developments in Watchdog 2.0. New features are broadly grouped into the categories reusability, reproducibility, and workflow execution. *Left:*New modules can now be developed in a semi-automated manner from software help pages using a GUI. A standardized documentation format was developed, allowing the automatic compilation of a reference book of available modules. Public repositories for sharing modules and workflows are now available. *Center:* Extensive logging of workflow execution ensures reproducibility of results and allows automated creation of a step-by-step report on analysis methods. Versioning of modules allows adaption to new requirements with backward compatibility without unnecessary module duplication. Software and module versions are now automatically reported in the log files. Execution wrappers now allow automatic deployment of software using container virtualization or package managers. *Right:* Workflow execution becomes more flexible with the resume and detach/reattach modes. The resume mode allows the resumption of interrupted or modified workflow execution without unnecessarily rerunning tasks. Detach/reattach allows the scheduler to be shut down on the local host while tasks on a computer cluster continue running and reattaching to workflow execution on the same or a different computer at a later time.

In the following, we describe only new developments that were added in Watchdog 2.0. The general principle of Watchdog and features already present in the previously published version remain unchanged; thus, we refer the reader to our previous publication for a detailed introduction to Watchdog [12]. The central improvements provided by Watchdog  2.0 are the following and are described in more detail in subsequent sections (see Fig. 1 for an overview). First, Watchdog 2.0 now provides a GUI for semi-automatically creating a new module from a software’s help page. Second, a standardized documentation format for modules was introduced in Watchdog 2.0. From module documentation files, a searchable module reference book can then be generated providing an overview and details on existing modules. Third, a community platform was created for sharing Watchdog modules and workflows with other scientists.

Improvements for reproducibility of analysis results comprise extensive logging of executed steps, including module and software versions, and the possibility to automatically generate a summary of the executed workflow steps, e.g., as a draft for an article methods section. In addition, we added fully integrated support for container virtualization or package managers in the form of so-called execution wrappers, in particular for Docker containers and the Conda package management system.

Finally, 2 additional execution modes were implemented to provide more comfort and flexibility in workflow execution. The resume mode allows execution of a workflow to be restarted by (re-)running only tasks that previously did not run (successfully) or were added or modified compared to the original execution. The second mode allows the scheduler to be detached from workflow execution without aborting tasks running on a computer cluster and reattaching to execution at a later time on the same or a different computer.

The GUI for module creation and all new command-line tools described in the following are implemented in Java and thus platform-independent.

### Semi-automated module generation

To make a software package available for use in Watchdog workflows, a new module has to be created. Watchdog already provides a helper script for creating the module XSD file and (optionally) a skeleton Bash script that only has to be extended by the program call. Nevertheless, this requires manually listing all parameters for the module. The newly developed GUI moduleMaker [16] now automatically extracts parameters and flags from a software help page to more conveniently create the corresponding module.

The moduleMaker GUI uses sets of regular expressions matching common help page formats to parse the help page of a software. Currently, 8 pre-defined regular expression sets are provided, but users can also define new sets using the GUI and add them to the pre-defined list. When creating a module with the GUI, users may either choose 1 particular regular expression set explicitly or let moduleMaker rank the regular expression sets based on how well they match the help page. In the latter case, the user can then examine the results of the *n* best-matching regular expression sets (with *n* user-defined) and choose the result they consider best. Subsequently, the user can correct errors in the automatic detection, add additional flags or parameters, and modify or delete detected parameters. In a next step, existence checks for input files or directories can be added and return values for the module can be defined.

Once the user is finished, moduleMaker creates the module XSD file and a wrapper Bash script for the software that—in contrast to the skeleton Bash script created by the helper script—is almost complete. The only manual changes required by the developer involve assigning values to return values. This wrapper script checks that required software is installed, parses parameters, verifies that mandatory parameters are set, performs existence checks on required input files and directories, executes the program, performs default error checks after execution, and writes return values to a corresponding file read by the scheduler. Optionally, a project file can be saved that allows modules created with the moduleMaker to be reloaded and modified at a later time.

Thus, developing a module does not require understanding XML or the module XSD schema. Furthermore, little or no Bash scripting experience is required if the GUI or helper script is used, respectively. The GUI creates a Bash script that is finished apart from the return value assignment. If the helper script is used, there is no requirement to use a Bash script to execute the commands. Any type of executable can be called in the module, e.g., a Python script. Examples for modules using Python scripts are included in the new module repository (see below).

### Module documentation

**Figure 2:**
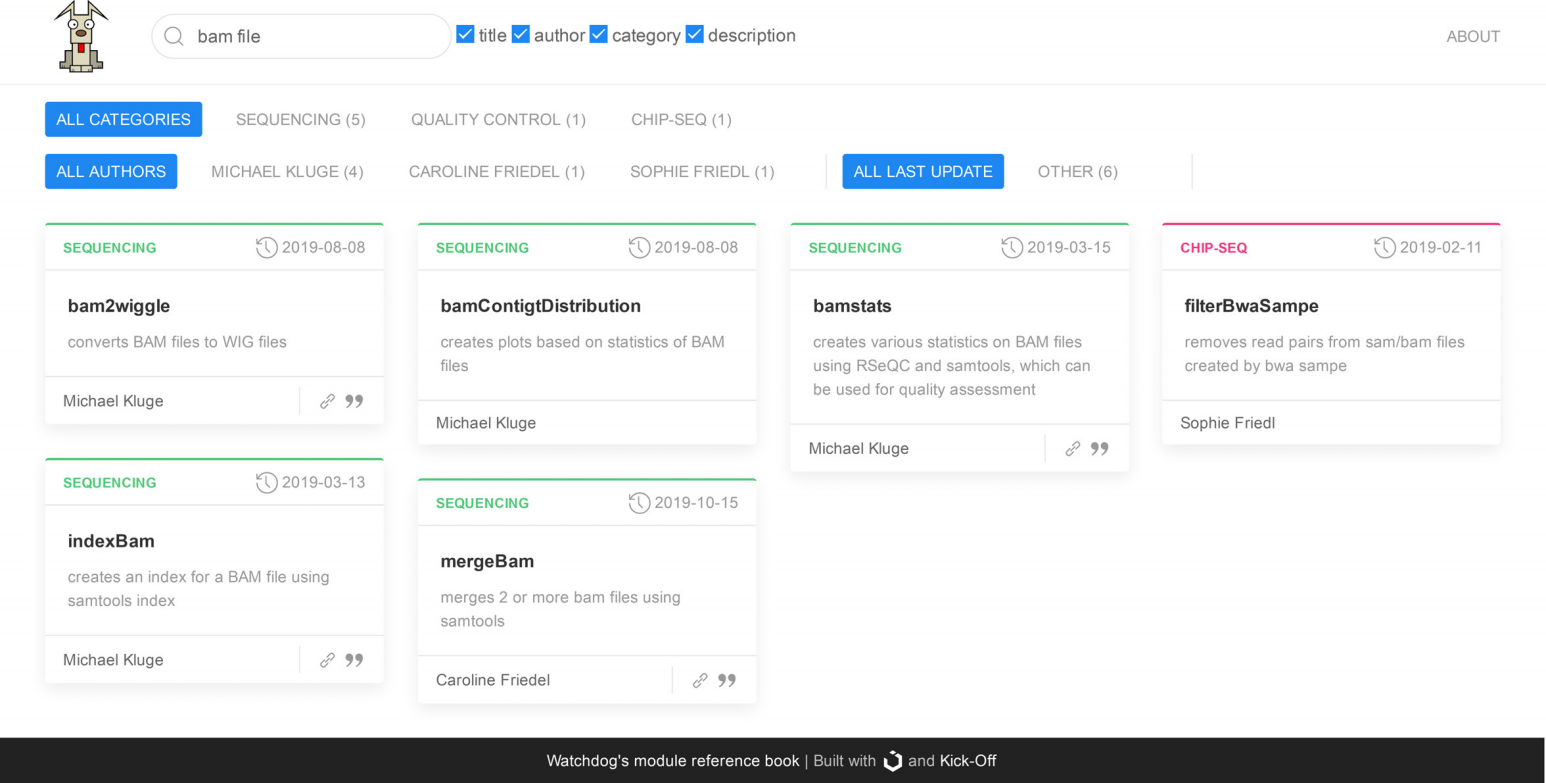
Overview page of the module reference book. The main section displays available modules as boxes, showing the module name, date of last change, a short description, links, and the author of the module. A search bar and category bar can be used to filter the displayed modules using text search or multi-category filters. In this example, all modules containing the term “bam file” in the description are shown.

While the Watchdog scheduler, features of Watchdog workflows, and workflow creation are already comprehensively documented [12], no convenient way was so far available for documenting both individual Watchdog modules and the set of available modules. To address this problem, we developed (i) a standardized documentation format for modules and (ii) a program for creating a nicely formatted, searchable, and updatable catalog of modules, the so-called reference book (see Fig. 2 for an example), from the documentation files of individual modules. The module entry in the reference book describes software dependencies, parameters (i.e., input files and values) and their default values, return values (i.e., output files and values), and more. Thus, instead of inspecting the module XSD or input mask in the GUI to obtain this information, users can now simply browse the reference book.

#### Documentation format

Individual Watchdog modules are now documented using a standardized XML format. This contains general module information (e.g., author, description, dependencies) and properties of module parameters and return values (e.g., name, type, description). The allowed semantic is described by an XSD schema file, allowing the XML documentation files to be read and further processed by XML parsing software.

To limit the overhead for creating the module documentation, a command-line tool (docuTemplateExtractor) is provided by Watchdog 2.0. The docuTemplateExtractor extracts parameter and return value information from the module XSD file and generates a template documentation file. Module developers then only have to fill in parts of the XML documentation not contained in the module XSD file.

As noted above, modules may also contain additional scripts, which can contain further information useful for documentation. For example, many scripts utilize an argument parser that requires a description or default values for each parameter. To exploit this and guarantee consistency between documentation and scripts, the docuTemplateExtractor also aims to extract this information. Because the syntax used by the argument parser strongly depends on both the scripting language used and the argument parser, this information cannot be obtained with a generalized approach. Instead, we developed a plugin system that allows developers to load custom parameter and return value extractors by implementing a simple Java interface. Currently, 2 parameter extractors for Bash- and Python-based modules are available, which obtain description and default value of parameters from argument parser definitions. For Bash scripts, the shFlags library is supported, and for Python, the argparse library.

#### Reference book

The reference book is implemented as an HTML web page based on the UIkit framework [17]. It can be opened with any browser supporting JavaScript and does not require a dedicated web server. The reference book can be created from the XML documentation files using the refBookGenerator command-line tool. The reference book can be created either for publicly available modules, personal modules of the user, or a combination of both. When new modules are added or existing modules are removed, the reference book can simply be regenerated using the refBookGenerator. Thus, every user can generate their personalized reference book containing the modules they work with or consider relevant to their work.

**Figure 3:**
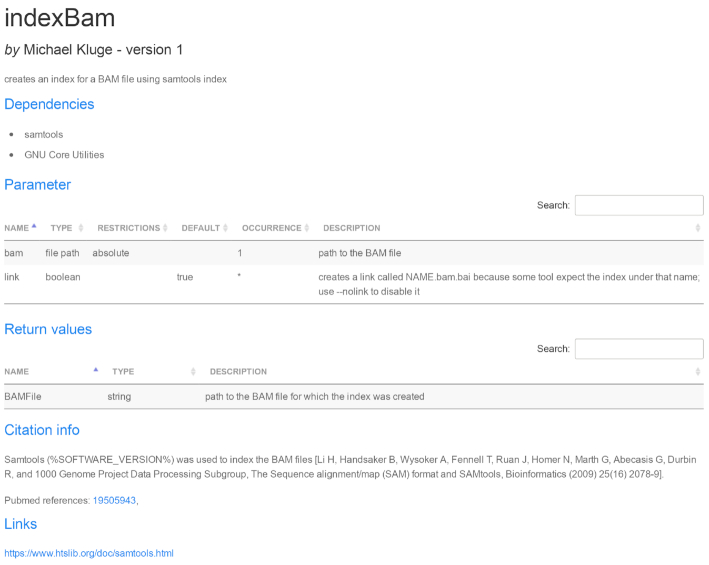
Detailed view of a module in the reference book. As an example, the detailed view on the indexBam module is shown, containing a short description, dependencies on third-party software, parameters with valid ranges and descriptions, return values, citation information, and web links. The citation information will also be included into the step-by-step report automatically created from the workflow execution log file.

Fig. 2 shows the front page of the module reference book (generated for all publicly available modules) after searching for modules containing the term “bam file” in the description. The main section of the front page provides an overview on all available modules. Every module is visualized as a box that contains its name, author, assigned category, and a short description. The search bar at the top can be used to filter modules using a keyword search, which can be applied to title, author, category, and/or description. Alternatively, the modules displayed in the overview section can be filtered on the basis of authorship, category, and update date. Clicking on a module box opens a detailed view, showing module dependencies, parameters and valid input values, return values, and if applicable citation information and web links (see Fig. 3 for an example).

### Public repositories for module and workflow sharing

Watchdog 2.0 now provides 2 repositories on Github under the watchdog-wms organization [18] that are dedicated for sharing modules [19] and workflows [20], respectively, by other users. To contribute either a module or workflow to one of the repositories, users have to first create a copy (fork) of the repository, change or add modules/workflows, commit the proposed changes to the repository copy, and submit these changes for review to the original repository via a pull request. An integration pipeline then checks whether the proposed changes adhere to essential requirements. If all automatic tests were successful, the proposed changes can be accepted by Watchdog team members.

Currently, the module repository contains 60 modules. Each module is located in a separate directory and must contain at least the XSD module file and an XML documentation file. Currently, most available modules focus on sequencing data analysis, in particular RNA-seq and ChIP-seq analysis. Some modules provide basic functionalities like file compression or text search while others fulfill more specific tasks, e.g., differential gene expression analysis (module DETest), peak detection in ChIP-seq data (module GEM), or identification of circular RNAs (modules circRNAfinder and ciri2). By default, modules are licensed under Apache License 2.0, but a different license can be assigned to a module by including it in the module folder. A reference book for all modules in the repository is available [21]. It is automatically updated with every commit to the master branch of the module repository.

Workflows shared in the watchdog-wms-workflows repository also have to be located in separate directories. Each workflow directory has to contain the XML workflow file, a readme file, and optionally example data. Workflows should be documented with inline comments. Furthermore, lines that require modifications to adapt, e.g., to different computing environments or input data should be highlighted to allow everyone to quickly adapt the workflow. We recommend, but do not enforce, that paths or constant parameter values are not hard-coded in the task section of the workflow, but rather that global constants are defined in the settings section. A constant "CONSTANT" can then be referenced as "${CONSTANT}" within process block or task definitions. If this recommendation is followed, the workflow can be quickly adapted to a new environment or data by modifying only constants and executors.

Currently, the workflow repository contains, e.g., the workflow for RNA-seq mapping and differential gene expression analysis from the original Watchdog release. Additionally, new workflows are available, e.g., for circular RNA detection with CIRI2 [22] and circRNA_finder [23], ChIP-seq analysis using GEM (GEM, RRID:SCR_005339) followed by ChIPseeker [24,25], and download of public NGS data from the NCBI SRA (SRA, RRID:SCR_004891) [26] followed by alignment with HISAT2 (HISAT2, RRID:SCR_015530) [27].

### Methods for ensuring reproducibility

A critical aspect of any analysis of biological data is the reproducibility of the results. While the use of a WMS already contributes to reproducibility, workflows may be modified between different runs of the workflow, e.g., by changing parameter values or including or excluding some steps, or the underlying software may be changed, e.g., by updates to a new version. This may lead to uncertainty regarding the steps, parameters, and software environment of the analysis that produced specific results. Furthermore, when reporting the individual steps of an analysis, for instance in a publication, some steps may be unintentionally omitted, making it difficult for others to reproduce the results. To address these problems, Watchdog 2.0 includes a number of new developments to ensure reproducibility of analyses.

#### Logging and automated reporting

When executing a workflow, Watchdog 2.0 now produces a time-stamped log file (filename extension .resume) reporting on the successful execution of each individual task. This log file is also used for the resume mode (see below). If a task creates multiple subtasks, e.g., for multiple input samples, successful execution of each subtask is recorded. For each task/subtask the log file records the value of each input parameter, as well as return values.

**Figure 4:**
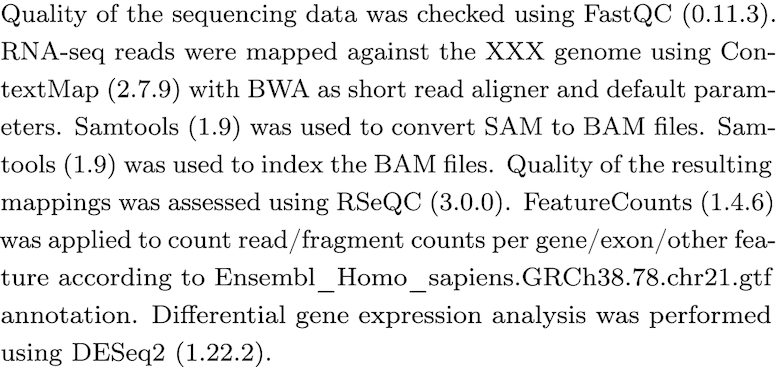
Result of automated report generation for example workflow. This example shows the step-by-step analysis report generated with the reportGenerator from the execution log file for the RNA-seq example workflow provided with Watchdog. The workflow was described in detail in our original Watchdog publication [12]. The annotation file name (a parameter to featureCounts) and software version numbers in parentheses are automatically obtained from the log file (see software version logging). For this example, the workflow was simplified to perform differential gene expression analysis only with DESeq2, instead of 4 different gene expression analysis methods as previously described. For modules without paper description (e.g., unzipping or replicate merging), the report would contain the text “No short description given in documentation of module *module name*”. To shorten this example, these sentences were manually removed, as well as the citation information commonly included in the module descriptions.

Moreover, a report of the executed steps can be automatically created from the log file using the new command-line tool reportGenerator provided with Watchdog 2.0 (see Fig. 4 for an example of the report). For this purpose, the XML documentation file of each module contains the element paperDescription, which can be filled with a short description of the module and citation information. It can also contain references to parameters of the task or the software version (see below for software version logging). The reportGenerator concatenates these descriptions in the order the corresponding tasks were executed and replaces references by the values reported in the log file. There is also an option to include PubMed IDs from the module documentation. The resulting report can then be used as a step-by-step protocol of the analysis or be further revised for the methods section of an article.

#### Module versioning

Modules generally rely on third-party software that can be modified repeatedly to improve performance, fix bugs, or be adapted to changing requirements, for instance by adding support for new types of experimental data. As a consequence, a module will need to be adapted over time, e.g., by changing the parameters of the module to support new parameters or drop obsolete ones. At the same time, backward compatibility needs to be ensured such that previously defined workflows relying on the old module version can still be executed. One solution to this problem would be to duplicate the module and adapt the copy. However, this leads to unnecessary code duplication because most of the module XSD file will remain unchanged, and results in code that is difficult to maintain.

To avoid this problem, Watchdog 2.0 now allows different versions of a module within 1 module XSD file to be defined by specifying the minimum and maximum supported module version for each element in the XSD file. If neither minimum nor maximum supported version is indicated, the element is valid for all module versions. This allows input parameters, return values, or even the executed program call to be changed between different module versions. When executing a workflow, the module version for each task will also be recorded in the log file. By default, the first version of a module is used unless otherwise specified in the workflow XML file. This guarantees that workflows defined before a new module version was introduced do not have to be adapted.

#### Software version logging

Watchdog is very flexible with regard to how dependencies to third-party software in a module can be handled by module developers. Software can be shipped with the module, loaded via package and environment management systems like Conda [13], or be required to be installed on the system that will execute the corresponding task (e.g., the local host or a computer cluster). In any case, it is crucial to know which software versions were run for a particular analysis in order to reproduce the analysis results or understand differences in outputs between repeated runs because new software releases often correct errors or may change the behavior of the software.

Thus, Watchdog 2.0 now implements a general approach for reporting versions of third-party software used in a module in the log file. For this purpose, a new attribute in the module XSD file can be used to define the flag for version printing of third-party software. During workflow execution, after a task or subtask has been completed successfully on a particular computer, the program call defined in the corresponding module is invoked with the version flag on the same computer to retrieve the installed third-party software version. This software version is then reported for the task/subtask in the log file. If the version flag has not been defined in the module, this step is omitted for the corresponding tasks. This option is also useful for identifying differences in installed third-party software between different executors used for workflow execution, such as the local host, a computer cluster, or remote executors accessed by SSH.

#### Execution wrappers

A disadvantage of Watchdog’s flexibility on how installation of third-party software is handled is that it complicates both reusability and reproducibility of workflows. Having to install all required software before modules or workflows can be used can be cumbersome. Furthermore, to fully reproduce results from a workflow, users would have to make sure that they (still) have the same software versions installed as in the original run of a workflow. Thus, we now implemented execution wrappers to explicitly support automatic deployment of software via package managers or container virtualization in Watchdog 2.0. Execution wrappers are initialized in the settings section of a Watchdog workflow and are then assigned to individual executors, which in turn use the wrapper to deploy the software for all tasks they run. Each executor can be assigned both a package manager and a container; thus, package managers can also be used within containers. Furthermore, different packager managers or containers can be assigned to different tasks by using different executors and corresponding execution wrappers for these tasks. Execution wrappers are implemented using Watchdog’s plugin system; thus, the set of available execution wrappers can be extended by users without having to modify the Watchdog code.

Currently, Watchdog  2.0 provides execution wrappers for the Conda package manager (Conda, RRID:SCR_018317) [13] and for Docker container virtualization [14]. To enable use of Conda for a module, the module directory only has to contain a YAML file defining the default Conda environment (modulename.conda.yml). For different versions of a module, different Conda environments can be defined (ending in .v[0-9]+.conda.yml). If no version-specific Conda definition file is found, the default Conda environment for the module is used. If Conda execution wrappers are not used in a workflow or for a particular executor, the Conda environment definition will simply be ignored for the whole workflow or the tasks run by the executor, respectively. Thus, previously developed workflows will not be affected by these changes.

The Docker execution wrapper allows tasks to be run within containers built from Docker images using Docker, Podman, or Singularity. Furthermore, it provides an option for automatically mounting files and directories on the host machine that are used in parameters of tasks. This option is enabled by default but can be disabled. Thus, adding container virtualization to an executor does not require changes to corresponding tasks. Similar to the Conda execution manager, module- and module-version-specific Docker images can be enabled by adding 1 or more files to the module folder specifying the image name to be used for the corresponding tasks. An example for using Docker and Conda in combination is provided in the workflow for RNA-seq mapping and differential gene expression analysis available from the workflow repository and with the Watchdog distribution.

### New execution modes

**Figure 5:**
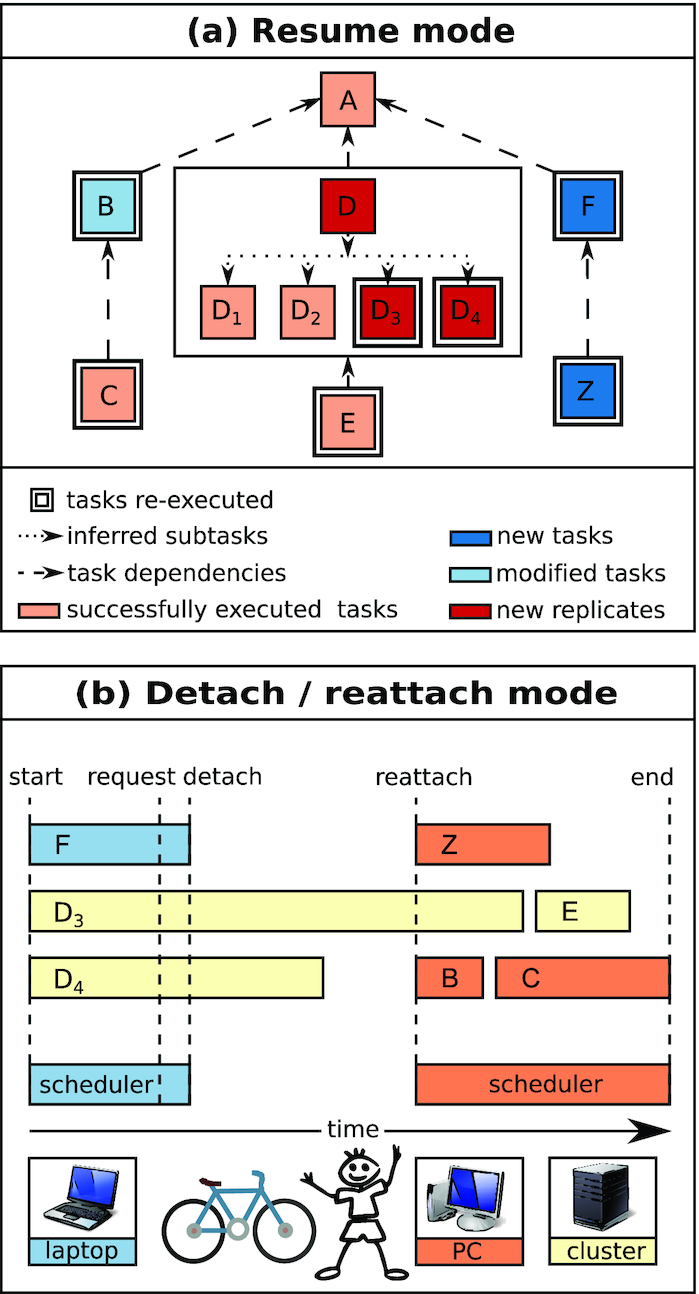
New execution modes in Watchdog 2.0. **(a)** Resume mode: From the log file of a previous workflow execution and the workflow XML file, the Watchdog scheduler automatically detects (sub)tasks that either have not yet run successfully, are new or modified, or require processing of additional samples. Consequently, only these (sub)tasks are executed, as well as all (sub)tasks depending on them (dependencies indicated as dashed lines). Light red indicates (sub)tasks that were previously executed successfully; light blue, tasks that were since modified; dark blue, new tasks that were added; and dark red, additional subtasks that have to be executed because additional samples were added. Double lines around tasks indicate which (sub)tasks have to be (re-)executed after resuming this workflow. **(b)** Visualization of the detach/reattach mode after resuming the workflow shown in (a). In this case, subtasks of D and task E are executed on a computer cluster, while a local executor is used for all other tasks. In this example, the Watchdog scheduler is originally started on a laptop and some tasks are scheduled and executed. After a while, a detach request is sent and no more new tasks are scheduled on the local host. Once the tasks on the local computer (blue) have finished, the detach file is written and the scheduler terminates. Subtasks D_3_ and D_4_ submitted to the computer cluster (yellow) continue to be executed. When the user reattaches to workflow execution, this time on a desktop computer (orange), new tasks are again scheduled.

In the original Watchdog version, the Watchdog scheduler had to run continuously on the computer on which workflow execution is started. If workflow execution was interrupted, e.g., by a computer crash or reboot, only a manual restart option was available. This required the last task finished successfully to be identified or some analyses to be rerun in case only some subtasks of a task finished successfully. To avoid this problem, Watchdog 2.0 now supports 2 additional execution modes (see Fig. 5). The first one allows workflow execution to be resumed at any point and rerunning only the tasks or subtasks in a workflow that did not finish successfully, were modified, or depended on modified tasks. The second execution mode allows detachment from workflow execution by shutting down the Watchdog scheduler on the current computer while tasks distributed to a computer cluster continue running. The scheduler can then reattach to the workflow execution at a later time either from the same or a different computer. This can be used for instance to reboot the machine running the scheduler or to switch from a desktop computer to a laptop without interrupting execution of tasks running on a computer cluster.

#### Resume mode

As described above, Watchdog 2.0 creates a detailed log file during execution of a workflow containing successfully finished (sub)tasks, as well as their input parameters and return values. In resume mode, Watchdog 2.0 uses the log file of a previous workflow run to determine which (sub)tasks have to be (re-)executed. Individual (sub)tasks are identified by their input parameter combinations. (Sub)tasks not listed in the log file with exactly the same input parameter values will be scheduled to be executed. Furthermore, (sub)tasks that previously finished successfully with the same parameters are re-executed if they depend on other (sub)tasks that are (re-)run.

This allows the resumption of not only workflows that were interrupted unexpectedly (e.g., by hardware failure or power outage) but also workflows that were modified, i.e., by changing parameters for some tasks, without unnecessarily rerunning tasks. Here, Watchdog 2.0 guarantees that all results are updated that may be affected by the modification. Furthermore, additional samples, e.g., for other conditions or more replicates, can be easily included without rerunning analyses for samples that have already been processed. Importantly, identification of (sub)tasks that require (re-)execution is performed automatically without manual user input. This both reduces the overhead for the user and eliminates the risk that they may forget some steps that need to be repeated.

The Watchdog 2.0 resume mode is illustrated in Fig. 5a for an example workflow. In this case, a task was modified (task B); additional samples were added to task D (marked red), requiring additional subtasks to be run; and additional tasks were added (F and Z). In resume mode, task B will be rerun because of modified parameters and task C because it depends on task B. For task D, only the new subtasks will be executed, but task E will be repeated because it depends on D. In addition, the newly added tasks will be run.

It should be noted here that after a workflow has been run at least once, changes to the workflow should be limited to adding new (sub)tasks (e.g., for new samples) or dependencies. Removing (sub)tasks or dependencies between tasks may lead to inconsistencies with old versions of input data being accidentally used for a task. Thus, this should only be done with utmost care.

#### Detach / reattach mode

In most cases, the Watchdog scheduler will run on a laptop or desktop computer and outsource all resource-intensive tasks to a distributed computer system, e.g., a computer cluster. Because execution of long, resource-intensive workflows may take hours or even days to complete, it may not always be possible for the Watchdog scheduler to be running continuously on the host computer. For instance, the host running Watchdog might require a reboot to install software updates or dedicated computer cluster submission hosts may not allow long-running programs. If the Watchdog scheduler is run on a laptop, the user may want to change locations with their laptop. To support these use cases, Watchdog 2.0 now provides the option to detach the scheduler from a running workflow and reattach at a later time. Notably, the user does not have to decide before execution whether to use this mode but can decide to detach at any time after starting execution in either normal or resume mode.

In Watchdog 2.0, the user can request to detach using a keystroke combination (Ctrl-C) or a link in the email notification. After the request is sent, Watchdog will wait for tasks to complete that are running either on the local host or a remote host via SSH, but schedule no further tasks on these executors. In contrast, Watchdog will continue to submit tasks on cluster executors with workload managers working independently of Watchdog (currently SGE and SLURM are supported). Once all tasks on the local and remote hosts are finished, Watchdog will save the information on tasks running on cluster executors to a file and then terminate itself. From this moment, tasks already running on or submitted to computing clusters will continue running or be scheduled to run by the corresponding workload managers, but no new tasks can be submitted to these clusters.

The detach file can then be used at a later time to reattach to workflow execution at the point where it was stopped previously. Watchdog will then obtain information on the execution status of tasks that were still running on or submitted to computer clusters before detaching, i.e., whether they are still running or finished successfully or with errors, and continue scheduling tasks on all executors accordingly. Notably, the Watchdog scheduler can also be reattached on another computer using the detach file, allowing for instance switching from a laptop at home to a desktop computer at work as illustrated in Fig. 5b. Moreover, Watchdog 2.0 also provides a command-line tool to periodically start the scheduler in auto-detach mode. In this mode, the scheduler checks whether tasks were finished successfully, submits new tasks if possible, and then terminates itself automatically.

### Comparison to other WMSs

In this article, we present a number of new developments in our WMS Watchdog. The previously published version of Watchdog [12] was already extensively compared against the most popular WMSs for biological analyses, i.e., Galaxy (Galaxy, RRID:SCR_006281) [8], KNIME (KNIME, RRID:SCR_006164) [9], Snakemake (Snakemake, RRID:SCR_003475) [10], and Nextflow [11] (see Fig. 12 in [12] for this comparison). Compared features included, e.g., availability of GUIs/web interfaces for workflow design, execution and monitoring, support for parallel, distributed, and cloud computing, dependency definition, and many more. This showed that Watchdog combined features of existing WMSs and provided novel useful features for execution and monitoring of workflows for users both with and without programming skills.

Because these features are essentially unchanged, we do not repeat this comparison here but refer the reader to our original publication [12]. In the following, we discuss how Watchdog 2.0 compares to these other WMSs regarding the new features we present in this article because these were not previously analyzed. First, we provide a brief description of Galaxy, KNIME, Snakemake, and Nextflow. For more details, see our original publication [12].

Galaxy is targeted at experimentalists without programming experience and allows data analyses to be performed in the web browser. Workflows can be constructed on public or private Galaxy servers in a web-based user interface from a set of available tools and can then be executed. New tools for use in a Galaxy workflow are defined in an XML format specifying the input parameters for this tool, as well as the program to execute.

KNIME is an open-source data analysis platform based on the Eclipse integrated development environment (IDE). It provides a powerful GUI for workflow construction, execution, and visualization of results, which can also be used without programming experience. Java programming skills are required for making a new tool available in a so-called node because multiple Java classes have to be extended.

Snakemake uses a Python-based language to define workflows in a so-called Snakefile as a set of rules that describe how output files are created from input files. Dependencies between rules are determined automatically on the basis of input and output files, and the order of rule execution is determined upon invocation based on these dependencies. Encapsulation of reusable components can be performed using so-called wrappers. Writing workflows and wrappers requires knowledge of the Snakemake syntax and some degree of programming skills.

Nextflow extends the Unix pipes model to transfer complex data between consecutive processes as shared data streams. It provides its own scripting language based on the Groovy programming language to define workflows. Individual analysis steps are defined as processes in the Nextflow workflow itself; thus, no actual encapsulation of tools into reusable components is supported. Similar to Snakemake, programming experience is required to define workflows and no GUI is provided.

For the following comparison, features were grouped broadly into categories reusability, reproducibility, and workflow execution. A summary of the comparison is presented in Table 1.

**Table 1:** Comparison of Watchdog with 4 other commonly used WMSs.

Category	No.	Feature	Watchdog	Galaxy	KNIME	Snakemake	Nextflow
Reusability	F1	Support for tool creation	Command-line/GUI	Command-line^1^	Eclipse Wizard	No	NA
	F2	Tool documentation	XML based	XML based	XML based	YAML based^2^	NA
	F3	Tool reference book	Web page generator	Part of GUI	Part of GUI	Web page generator	NA
	F4	Tool versioning	Yes	Yes	Yes	Yes	NA
	F5	Sharing of tools	Repository^3^	ToolShed^4^	KNIME Hub^5^/NodePit^6,*^	Repository^7^	NA
	F6	Sharing of workflows	Repository^3^	ToolShed^4^	KNIME Hub^5^/NodePit^6,*^	Repository^8^	nf-core^9,*^
	F7	Repurposing of workflows	XML edit/GUI	GUI	GUI	Copy Snakefile	Command-line
Reproducibility	F8	Software version logging	Yes	No	No	Yes	No
	F9	Software deployment	Execution wrappers, Conda, Docker	Conda, Docker	No	Conda, Docker	Conda, Docker
	F10	Creation of workflow report	Yes	List via history	Static description^10^	HTML report	HTML report
	F11	Citation export	Yes	Yes	No	No	No
Execution	F12	Resume workflow	Yes	No	Yes	Yes	Yes
	F13	Process only updated tasks	Yes	No	Yes	Yes^11^	Yes
	F14	Process only new replicates	Yes	No	No	Yes^11^	Yes
	F15	Detach/reattach	Yes	Yes^12^	Non-free feature^13^	Yes^14^	No

The selected WMSs are compared against Watchdog based on features grouped broadly into the categories reusability, reproducibility, and execution. NA: not applicable. ^1^Python-based command-line program (Planemo). ^2^No explicit documentation of parameters but example Snakefile and wrapper source code is part of the documentation. ^3^[18]. ^4^[28]. ^5^[29]. ^6^[30]. ^7^[31]. ^8^[32]. ^9^[33.] ^10^A description that was manually created for a specific workflow can be displayed but is not dynamically created. ^11^Flag "--list-params-changes" or "--list-input-changes" in combination with the "--forcerun" flag. ^12^Client: anytime/server: if jobs are not executed locally on the server. ^13^Non-free SGE extension or KNIME server required. ^14^Sending of a TERM signal stops scheduling of new jobs and waits for all running jobs to finish; Ctrl+C kills all jobs running on the local computer, while jobs running on a computing cluster continue to run. *Community project.

#### Reusability

For this part of the comparison, we focused on features that support development and sharing of tools (modules in Watchdog, tools in Galaxy, nodes in KNIME, rules in Snakemake, processes in Nextflow) for (re-)use in multiple analysis workflows as well as sharing and repurposing of existing workflows (F1–F7 in Table 1). Because there is no real encapsulation of tools in Nextflow, most of these features are not applicable to it.

Support for tool creation (F1) is provided in Galaxy by the command-line program Planemo, which is similar to the helper script originally provided by Watchdog for module creation. Notably, Planemo also requires all parameters for a new tool to be added manually. For KNIME, an Eclipse extension (KNIME Node Wizard) is available, which generates the project structure, the plug-in manifest, and all required Java classes. However, the Java classes only contain the basic backbone (in particular, no parameters or flags) and have to be massively extended by the developer. Snakemake does not provide any software or script for defining wrappers.

All 3 WMSs allow documenting (F2) tools and their parameters in XML or YAML format. In the case of Snakemake, the specification does not require explicit documentation of parameters and input and output. Instead, an example Snakefile showing the use of the wrapper has to be provided. A reference book containing information on all available tools (F3) can be generated for Snakemake wrappers as a separate web page. This contains the example Snakefile, the code of the wrapper, author information, and software dependencies. In contrast, the documentation of KNIME nodes and Galaxy tools, respectively, is displayed on their respective GUI/web interface during workflow creation. Furthermore, all 3 WMSs perform tool versioning (F4).

For sharing tools (F5) or complete workflows (F6) with other users, Galaxy and KNIME operate dedicated sharing platforms [28,29], while Snakemake provides source code repositories similar to Watchdog 2.0 [31,32]. Furthermore, dedicated sharing platforms are operated by the KNIME and Nextflow community [30,33].

Repurposing an existing workflow for new data (F7) requires different steps in the different WMSs. In Galaxy and KNIME, existing workflows can be imported and subsequently input files or values have to be selected/modified in the web interface and GUI, respectively. For Nextflow, input is provided via command-line parameters. For Snakemake, relative paths to input files are hard-coded in the Snakefile. Thus, repurposing a Snakemake workflow only requires the Snakefile to be copied to a directory in which input files are stored or linked in the subdirectory structure used in the Snakefile. In Watchdog workflows, input files and parameters are also hard-coded but absolute paths are used. In a well-designed workflow, global constants are defined for input values and files in the settings section and used throughout the workflow. Thus, repurposing only requires these constants to be edited either in a text or XML editor or the GUI. This is not more effort than required by other WMSs, with the exception of Snakemake. However, it provides more flexibility than Snakemake regarding how input data are distributed in the file system, and workflows can be stored anywhere, e.g., in a directory containing all previously developed workflows.

#### Reproducibility

Here, we focus on features (F8–F11) related to reproducibility of analysis results carried out at an earlier time, on different computer systems, and/or by other scientists. Most of the other WMSs do not support explicit logging of external software during workflow execution similar to Watchdog 2.0 (F8). However, Galaxy, Snakemake, and Nextflow support controlling external software dependencies and versions with the Conda package manager or using Docker containers (F9). Furthermore, Snakemake reports on executed workflows (see next paragraph) display the Conda environment for each task, including software versions.

A description of all performed analysis steps (F10) can be obtained in Snakemake and Nextflow through generation of HTML reports, in which individual steps are listed in a table format and in the case of Snakemake also visualized as a graph. Galaxy displays all executed tasks as a list in its analysis history. In contrast, KNIME supports only static workflow descriptions that have to be prepared by the workflow developer. The dynamic report created by Watchdog 2.0 from the execution log does not only list performed steps but includes short descriptions of each step prepared by module developers with citation information and (optionally) PubMed IDs (F11). The only other WMS allowing the declaration of citations for tools is Galaxy. In this case, a list containing citations for all tools used can be exported after executing a workflow in Galaxy. None of the other WMSs support creation of a step-by-step report for inclusion in a manuscript draft similar to Watchdog 2.0.

#### Execution

All WMSs except Galaxy can resume execution of partly executed workflows (F12) and are able to detect new tasks, modified tasks, or tasks with altered dependencies and consequently execute only these tasks (F13). With Snakemake and Nextflow, new samples (e.g., additional replicates) can be included in an analysis workflow without having to reprocess all samples (F14), but this option has to be forcibly triggered in Snakemake. This is not possible for KNIME workflows. One possibility to avoid unnecessary reprocessing in KNIME is to implement KNIME nodes that can detect whether the corresponding task was already executed successfully on a sample as done by Hastreiter et al. [34]. However, this adds additional overhead for node development.

Finally, similar execution modes to the detach/reattach mode of Watchdog 2.0 (F15) are at least partly supported by all compared WMSs apart from Nextflow. Because Galaxy is a web-based system, the user can log off (detach) and log in (reattach) at any time and from different client systems. Furthermore, the Galaxy server can also be restarted while tasks continue running on a computer cluster if no tasks are executed locally on the server. In KNIME, remote execution is only possible with non-free extensions like the KNIME Server or a cluster extension. If tasks are executed remotely using such an extension, the local KNIME instance can be detached and reattached to workflow execution. Finally, Snakemake provides the option to stop scheduling by sending the TERM signal and waiting for all jobs to be finished before terminating. Later, workflow execution can then simply be resumed. However, this mode also stops scheduling of jobs on computer clusters and waits for jobs running on computer clusters to be finished. Alternatively, Ctrl+C kills the main Snakemake process and all jobs running on the local computer, but jobs already running on a computing cluster keep running. With the correct use of profiles, it is then possible for the workflow to check the status of those jobs after a restart.

## Conclusion

In this article, we present the new developments in Watchdog 2.0, which focus on improving reusability of modules and workflows, reproducibility of analysis results, and convenience of workflow execution.

To simplify module development, we developed the moduleMaker GUI for semi-automatically creating a module for a software package by parsing its help page. Manual overhead for the module creator is then mostly limited to choosing the best regular expression set, validating and correcting automatically identified parameters, and adding additional parameters or return values considered necessary. Furthermore, we established public sharing repositories to support and encourage exchange of developed modules and workflows between scientists. Modules are now documented in a standardized documentation format, from which an HTML-based module reference book can automatically be created. The reference book provides an overview and details on available modules and can be easily regenerated to integrate new modules, e.g., modules created by other developers.

To guarantee reproducibility of workflow results, we introduced module versions and extensive logging of successfully executed steps including parameter values and third-party software versions. From the log file of a workflow execution, a report can then be automatically generated that serves both as a documentation of the analysis steps and as a starting point for drafting the corresponding methods section of a manuscript. This not only reduces the effort in creating a description of the analysis, it also prevents accidental omission of individual steps. In addition, Watchdog 2.0 now provides integrated support for automatic deployment of software, in particular with Conda or Docker, in the form of execution wrappers.

Finally, with the new resume and detach/reattach execution mode, convenience and flexibility of workflow execution is greatly enhanced in Watchdog 2.0. The resume mode not only implements the state of the art for WMSs that allows resumption of interrupted workflow execution, but automatically identifies and re-executes tasks with modified parameters or additional input samples as well as downstream tasks that depend on them. The detach/reattach mode allows shutting down the Watchdog scheduler on a local computer while jobs continue to be executed on computer clusters. The user can then reattach to workflow execution and resume scheduling of tasks at a later time and even from a different computer.

While many of the new features in Watchdog 2.0 are also present in other popular WMSs, none are implemented in all of them. Furthermore, even if these features are available in other WMSs, the implementations in Watchdog 2.0 often add additional capabilities, such as, e.g., the possibility to automatically generate a step-by-step report. Combined with the existing advantages of Watchdog highlighted in our original publication, we thus believe that Watchdog 2.0 will be of great benefit to users with a wide range of computer skills for performing large-scale bioinformatics analyses in a flexible and reproducible manner.

## Availability of Source Code and Requirements

- Project name: Watchdog 2.0
- Project home page: https://www.bio.ifi.lmu.de/watchdog
- Source code: https://github.com/klugem/watchdog, https://github.com/watchdog-wms
- Operating system(s): Platform independent
- Programming language: Java
- Other requirements: Java 11 or higher, JavaFX 11 or higher for the GUIs, individual requirements for modules
- License: GNU General Public License v3.0
- DOI: https://doi.org/10.5281/zenodo.3764538
- RRID:SCR_018355
- biotoolsID: biotools:watchdog

## Availability of Supporting Data and Materials

Snapshots of the Watchdog 2.0 code and the module and workfow repository used for this article are available in the GigaDB data repository [35].

## Abbreviations

ATAC-seq: assay for transposase-accessible chromatin using sequencing; ChIP-seq: chromatin immunoprecipitation sequencing; GUI: graphical user interface; IDE: integrated development environment; JSON: Javascript Object Notation; NCBI: National Center for Biotechnology Information; NGS: next-generation sequencing; RNA-seq: RNA sequencing; SSH: Secure Shell; SRA: Sequence Read Archive; WMS: workflow management system.

## Competing Interests

The authors declare that they have no competing interests.

## Funding

This work was supported by grants FR2938/7-1, FR2938/10-1, and CRC 1123 (Z2) from the Deutsche Forschungsgemeinschaft (DFG) to C.C.F.

## Authors' Contributions

M.K. developed the software and wrote the manuscript. M.-S.F. tested Watchdog 2.0 and implemented modules and the workflow for the analysis of circular RNAs in high-throughput sequencing data. A.L.M. implemented the moduleMaker GUI under supervision of C.C.F. and M.K. C.C.F. tested the software, helped in revising the manuscript, and supervised the project. All authors read and approved the final manuscript.

## Supplementary Material

giaa068_GIGA-D-19-00409_Original_Submission

giaa068_GIGA-D-19-00409_Revision_1

giaa068_GIGA-D-19-00409_Revision_2

giaa068_Response_to_Reviewer_Comments_Original_Submission

giaa068_Response_to_Reviewer_Comments_Revision_1

giaa068_Reviewer_1_Report_Original_Submission Tazro Ohta -- 1/2/2020 Reviewed

giaa068_Reviewer_2_Report_Original_Submission Jan Forster -- 1/31/2020 Reviewed

giaa068_Reviewer_2_Report_Revision_1 Jan Forster -- 5/12/2020 Reviewed

giaa068_Reviewer_3_Report_Original_Submission Stian Soiland-Reyes -- 2/13/2020 Reviewed

giaa068_Reviewer_3_Report_Revision_1 Stian Soiland-Reyes -- 5/19/2020 Reviewed
