# Supplementary material for: Watchdog 2.0: New developments for reusability, reproducibility, and workflow execution
Source: Gigascience. 2020 Jun 17;9(6):giaa068. doi: 10.1093/gigascience/giaa068 (PMC7298769; doi:10.1093/gigascience/giaa068)
Supplement: giaa068_GIGA-D-19-00409_Revision_1 [file giaa068_giga-d-19-00409_revision_1.pdf]

GigaScience

Watchdog 2.0: New developments for reusability, reproducibility and workflow execution

--Manuscript Draft--

**Manuscript Number:**	GIGA-D-19-00409R1
**Full Title:**	Watchdog 2.0: New developments for reusability, reproducibility and workflow execution
**Article Type:**	Technical Note
**Funding Information:**	Deutsche Forschungsgemeinschaft (FR2938/7-1) — Prof. Dr. Caroline C. Friedel<br>Deutsche Forschungsgemeinschaft (FR2938/10-1) — Prof. Dr. Caroline C. Friedel<br>Deutsche Forschungsgemeinschaft (CRC 1123 (Z2)) — Prof. Dr. Caroline C. Friedel
**Abstract:**	Background: Advances in high-throughput methods have brought new challenges for biological data analysis, often requiring many interdependent steps applied to a large number of samples. To address this challenge, workflow management systems, such as Watchdog, have been developed to support scientists in the (semi-)automated execution of large analysis workflows.<br><br>Implementation: Here, we present Watchdog 2.0, which implements new developments for module creation, reusability and documentation and for reproducibility of analyses and workflow execution. Developments include a graphical user interface for semi-automatic module creation from software help pages, sharing repositories for modules and workflows and a standardized module documentation format. The latter allows generation of a customized reference book of public and user-specific modules. Furthermore, extensive logging of workflow execution, module and software versions and explicit support for package managers and container virtualization now ensures reproducibility of results. A step-by-step analysis protocol generated from the log file may e.g. serve as a draft of a manuscript methods section. Finally, two new execution modes were implemented. One allows resuming workflow execution after interruption or modification without re-running successfully executed tasks not affected by changes. The second one allows detaching and reattaching to workflow execution on a local computer while tasks continue running on computer clusters.<br><br>Conclusions: Watchdog 2.0 provides several new developments that we believe to be of benefit for large-scale bioinformatics analysis and that are not completely covered by other competing workflow management systems. The software itself, module and workflow repositories, and a comprehensive documentation are freely available at https://www.bio.ifi.lmu.de/watchdog.
**Corresponding Author:**	Caroline C. Friedel<br>Ludwig-Maximilians-Universitat Munchen<br>München, GERMANY
**Corresponding Author Secondary Information:**	
**Corresponding Author's Institution:**	Ludwig-Maximilians-Universitat Munchen
**Corresponding Author's Secondary Institution:**	
**First Author:**	Michael Kluge
**First Author Secondary Information:**	
**Order of Authors:**	Michael Kluge<br>Marie-Sophie Friedl

Powered by Editorial Manager® and ProduXion Manager® from Aries Systems Corporation

	Amrei L. Menzel
	Caroline C. Friedel
**Order of Authors Secondary Information:**	
**Response to Reviewers:**	The response to reviewers was uploaded as a separate PDF as Supplementary Material to the submission.
**Additional Information:**	
**Question**	**Response**
Are you submitting this manuscript to a special series or article collection?	No
**Experimental design and statistics**<br><br>Full details of the experimental design and statistical methods used should be given in the Methods section, as detailed in our Minimum Standards Reporting Checklist. Information essential to interpreting the data presented should be made available in the figure legends.<br><br>Have you included all the information requested in your manuscript?	Yes
**Resources**<br><br>A description of all resources used, including antibodies, cell lines, animals and software tools, with enough information to allow them to be uniquely identified, should be included in the Methods section. Authors are strongly encouraged to cite Research Resource Identifiers (RRIDs) for antibodies, model organisms and tools, where possible.<br><br>Have you included the information requested as detailed in our Minimum Standards Reporting Checklist?	Yes
**Availability of data and materials**<br><br>All datasets and code on which the conclusions of the paper rely must be either included in your submission or deposited in publicly available repositories	Yes

Powered by Editorial Manager® and ProduXion Manager® from Aries Systems Corporation

(where available and ethically appropriate), referencing such data using a unique identifier in the references and in the “Availability of Data and Materials” section of your manuscript.<br><br>Have you have met the above requirement as detailed in our Minimum Standards Reporting Checklist?	

Manuscript
Click here to access/download;Manuscript;main.pdf

Placeholder for OUP logo oup.pdf

GigaScience, 20xx, 1–10

doi: xx.xxxx/xxxx
Manuscript in Preparation
Technical Note

TECHNICAL NOTE

Watchdog 2.0: New developments for reusability, reproducibility and workflow execution

Michael Kluge[1], Marie-Sophie Friedl[1], Amrei L. Menzel[1] and Caroline C. Friedel[1,*]

[1]Institut für Informatik, Ludwig-Maximilians-Universität München, Amalienstr. 17, Munich 80333, Germany

*caroline.friedel@bio.ifi.lmu.de

Abstract

Background: Advances in high-throughput methods have brought new challenges for biological data analysis, often requiring many interdependent steps applied to a large number of samples. To address this challenge, workflow management systems, such as *Watchdog*, have been developed to support scientists in the (semi-)automated execution of large analysis workflows.
Implementation: Here, we present *Watchdog* 2.0, which implements new developments for module creation, reusability and documentation and for reproducibility of analyses and workflow execution. Developments include a graphical user interface for semi-automatic module creation from software help pages, sharing repositories for modules and workflows and a standardized module documentation format. The latter allows generation of a customized reference book of public and user-specific modules. Furthermore, extensive logging of workflow execution, module and software versions and explicit support for package managers and container virtualization now ensures reproducibility of results. A step-by-step analysis protocol generated from the log file may e.g. serve as a draft of a manuscript methods section. Finally, two new execution modes were implemented. One allows resuming workflow execution after interruption or modification without re-running successfully executed tasks not affected by changes. The second one allows detaching and reattaching to workflow execution on a local computer while tasks continue running on computer clusters.
Conclusions: *Watchdog* 2.0 provides several new developments that we believe to be of benefit for large-scale bioinformatics analysis and that are not completely covered by other competing workflow management systems. The software itself, module and workflow repositories, and a comprehensive documentation are freely available at https://www.bio.ifi.lmu.de/watchdog.

Key words: Workflow management system; Bioinformatics; Automated biological data analysis; Next-generation sequencing; Reusability; Reproducibility; Open science tools;

Background

Due to improvements in sequencing technologies, sequencing costs have dropped massively in the last years [1]. While the first human genome sequence cost about $2.7 billion and took 13 years to complete [2], companies now offer genome sequencing to private customers using state-of-the-art next-generation-sequencing (NGS) technologies for less than $1000. In addition, other cellular properties can now be measured at large scale using NGS. This includes e.g. the expression of genes (RNA-seq) [3], protein binding to DNA (ChIP-seq) [4], open chromatin regions (ATAC-seq) [5] and many more.

As a consequence, data analysis has become more complex with new challenges for bioinformatics, often requiring multiple interdependent steps and integration of numerous replicates and several types of high-throughput data. Since manual execution of all required analysis steps is cumbersome, time-consuming and laborious to repeat, several tools have been developed for performing large-scale bioinformatics analyses. One group of tools consists of static analysis pipelines specifically designed for one application, e.g. transcriptome analysis [6, 7]. While these pipelines have the advantage that a particular analysis can be repeated without great effort, components of these analysis pipelines are often not easily reusable for other related applications. As an alternative, workflow management systems (WMSs) have been developed that support creation of such analysis pipelines (denoted as workflows in this context) from reusable components and allow (semi-)automated execution of these workflows. Popular WMSs are *Galaxy* [8], *KNIME* [9], *Snakemake* [10] and *Nextflow* [11] and differ in the implemented set

Compiled on: April 24, 2020.
Draft manuscript prepared by the author.

of features, target audience, the required training period, usage fees and more (for more details see the comparison in the first article on *Watchdog* [12] and at the end of this article).

Previously, we presented the WMS *Watchdog* for the distributed analysis of large-scale experimental data originating e.g. from NGS experiments [12]. The core features of Watchdog include straightforward processing of replicate data, support for and flexible combination of distributed computing or remote executors, customizable error detection, user notification on execution errors and manual user intervention. In *Watchdog*, reusable components are encapsulated within so-called modules, which are defined by an XSD file specifying the program to execute, input parameters and return values of the module. In addition, modules can contain scripts or compiled binaries that are invoked in the module. There are no restrictions on included software or on the programming language used in additional scripts. Modules may also deploy required software internally using Conda [13], Docker [14] or similar tools.

A *Watchdog* workflow is defined in an XML format and consists of a sequence of tasks and dependencies between tasks. Each task uses one module and the same task can be automatically run on multiple samples or with multiple parameter combinations using so-called process blocks. This creates several subtasks, one for each sample or parameter combination. A workflow can either be created manually using any XML editor or the *Watchdog* graphical user interface (GUI) for workflow construction. While XML may be more complex than e.g. YAML or JSON, it is widely used and numerous XML editors are available, e.g. plugins for Eclipse [15]. Furthermore, using the GUI requires neither understanding of XML nor programming skills and thus allows easy construction of workflows from a pre-defined set of modules. In this case, the only *Watchdog* syntax that has to be learned is how to reference variables.

Workflows can be executed using the *Watchdog* scheduler via a command-line interface or the GUI, which are both implemented in Java and thus platform-independent. The *Watchdog* scheduler continuously monitors the execution status of tasks and schedules new tasks or subtasks for execution if all tasks they depend on finished successfully. The execution status of tasks is reported to the user via standard output, a web interface that allows manual intervention and (optionally) email.

In the workflow, different executors can be specified for different tasks. Currently, three types of executors are supported (local host, remote host via SSH or computer clusters using SGE or SLURM). Thus, resource-intensive or long-running tasks can e.g. be submitted to a computer cluster while less demanding tasks may be executed on the local host. Furthermore, *Watchdog* provides a plugin system that allows users with programming skills to add new executor types, e.g. for cloud computing, without having to change the original *Watchdog* code (for details see [12]).

In this article, we present *Watchdog* 2.0, a new and improved version of *Watchdog* with several new developments for module creation and documentation, reusability of modules and workflows, reproducibility of analysis results as well as workflow execution.

Implementation

Overview

In the following, we describe only new developments that were added in *Watchdog* 2.0. The general principle of *Watchdog* and features already present in the previously published version remain unchanged, thus we refer to our previous publication for a detailed introduction to *Watchdog* [12]. The central improvements provided by *Watchdog* 2.0 are the following and are described in more detail in subsequent sections (see Fig. 1 for an overview). First, *Watchdog* 2.0 now provides a GUI for semi-automatically creating a new module from a software's help page. Second, a standardized documentation format for modules was introduced in *Watchdog* 2.0. From module documentation files, a searchable module reference book can then be generated providing an overview and details on existing modules. Third, a community platform was created for sharing *Watchdog* modules and workflows with other scientists.

Improvements for reproducibility of analysis results comprise extensive logging of executed steps, including module and software versions, and the possibility to automatically generate a summary of the executed workflow steps, e.g. as a draft for an article methods section. In addition, we added fully integrated support for container virtualization or package managers in the form of so-called *execution wrappers*, in particular for Docker containers and the Conda package management system.

Finally, two additional execution modes were implemented to provide more comfort and flexibility in workflow execution. The resume mode allows restarting execution of a workflow by (re-)running only tasks that previously did not run (successfully), were added or modified compared to the original execution. The second mode allows detaching the scheduler from workflow execution without aborting tasks running on a computer cluster and reattaching to execution at a later time on the same or a different computer.

The GUI for module creation and all new command-line tools described in the following are implemented in Java and thus platform-independent.

Semi-automated module generation

To make a software available for use in *Watchdog* workflows, a new module has to be created. *Watchdog* already provides a helper script for creating the module XSD file and (optionally) a skeleton Bash script that only has to be extended by the program call. Nevertheless, this requires manually listing all parameters for the module. The newly developed GUI *moduleMaker* (available at https://github.com/watchdog-wms/moduleMaker) now automatically extracts parameters and flags from a software help page to more conveniently create the corresponding module.

The *moduleMaker* GUI uses sets of regular expressions matching common help page formats to parse the help page of a software. Currently, 8 pre-defined regular expression sets are provided but users can also define new sets using the GUI and add them to the pre-defined list. When creating a module with the GUI, users may either choose one particular regular expression set explicitly or let *moduleMaker* rank the regular expression sets based on how well they match the help page. In the later case, the user can then examine the results of the n best-matching regular expression sets (with n user-defined) and choose the result they consider best. Subsequently, the user can correct errors in the automatic detection, add additional flags or parameters and modify or delete detected parameters. In a next step, existence checks for input files or directories can be added and return values for the module can be defined.

Once the user is finished, *moduleMaker* creates the module XSD file and a wrapper Bash script for the software that – in contrast to the skeleton Bash script created by the helper script – is almost complete. The only manual changes required by the developer involve assigning values to return values. This wrapper script checks that required software is installed, parses parameters, verifies that mandatory parameters are set, performs existence checks on required input files and directories, executes the program, performs default error checks after execution and writes return values to a corresponding file read by the scheduler. Optionally, a project file can be saved that allows reloading and modifying modules created with the *moduleMaker* at a later time.

Thus, developing a module does not require understanding XML or the module XSD schema. Furthermore, little or no Bash scripting experience is required if the GUI or helper script is used, respectively. The GUI creates a Bash script that is finished apart from the return value assignment. If the helper script is used, there is no requirement to use a Bash script to execute the commands. Any type of

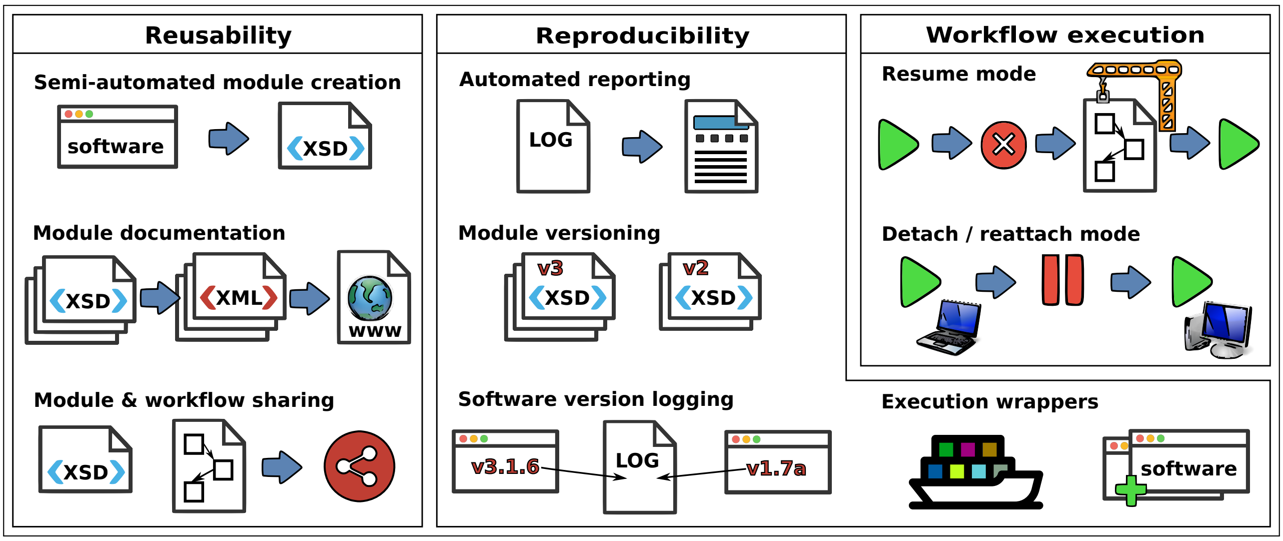

Figure 1. Overview on new developments in *Watchdog* 2.0. New features are broadly grouped into the categories reusability, reproducibility, and workflow execution. Left: New modules can now be developed in a semi-automated manner from software help pages using a GUI. A standardized documentation format was developed, allowing to automatically compile a reference book of available modules. Public repositories for sharing modules and workflows are now available. Center: Extensive logging of workflow execution ensures reproducibility of results and allows automated creation of a step-by-step report on analysis methods. Versioning of modules allows adaption to new requirements with backward compatibility without unnecessary module duplication. Software and module versions are now automatically reported in the log files. Execution wrappers now allow automatic deployment of software using container virtualization or package managers. Right: Workflow execution becomes more flexible with the resume and detach/reattach modes. The resume mode allows resuming interrupted or modified workflow execution without unnecessarily re-running tasks. Detach/reattach allows shutting down the scheduler on the local host while still running tasks on a computer cluster and reattaching to workflow execution on the same or different computer at a later time.

executable can be called in the module, e.g. a Python script. Examples for modules using Python scripts are included in the new module repository (see below).

Module documentation

While the *Watchdog* scheduler, features of *Watchdog* workflows and workflow creation are already comprehensively documented [12], no convenient way was so far available for documenting both individual *Watchdog* modules and the set of available modules. To address this problem, we developed i) a standardized documentation format for modules and ii) a program for creating a nicely formatted, searchable and updatable catalog of modules, the so-called reference book (see Fig. 2 for an example), from the documentation files of individual modules. The module entry in the reference book describes software dependencies, parameters (i.e. input files and values) and their default values, return values (i.e. output files and values) and more. Thus, instead of inspecting the module XSD or input mask in the GUI to obtain this information, users can now simply browse the reference book.

Documentation format

Individual *Watchdog* modules are now documented using a standardized XML format. This contains general module information (e.g. author, description, dependencies) and properties of module parameters and return values (e.g. name, type, description). The allowed semantic is described by an XSD schema file, allowing the XML documentation files to be read and further processed by XML parsing software.

To limit the overhead for creating the module documentation, a command-line tool (*docuTemplateExtractor*) is provided by *Watchdog* 2.0. The *docuTemplateExtractor* extracts parameter and return value information from the module XSD file and generates a template documentation file. Module developers then only have to fill in parts of the XML documentation not contained in the module XSD file.

As noted above, modules may also contain additional scripts, which can contain further information useful for documentation. For example, many scripts utilize an argument parser that requires a description or default values for each parameter. To exploit this and guarantee consistency between documentation and scripts, the *docuTemplateExtractor* also aims to extract this information. Since the syntax used by the argument parser strongly depends on both the used scripting language and the argument parser, this information cannot be obtained with a generalized approach. Instead, we developed a plugin system that allows developers to load custom parameter and return value extractors by implementing a simple Java interface. Currently, two parameter extractors for Bash- and Python-based modules are available, which obtain description and default value of parameters from argument parser definitions. For Bash scripts, the *shFlags* library is supported and for Python the *argparse* library.

Reference book

The reference book is implemented as an HTML webpage based on the *UIkit* framework [16]. It can be opened with any browser supporting JavaScript and does not require a dedicated web server. The reference book can be created from the XML documentation files using the *refBookGenerator* command-line tool. The reference book can be created either for publicly available modules, personal modules of the user or a combination of both. When new modules are added or existing modules are removed, the reference book can simply be regenerated using the *refBookGenerator*. Thus, every user can generate their personalized reference book containing the modules they work with or consider relevant to their work.

Fig. 2 shows the front page of the module reference book (generated for all publicly available modules) after searching for modules containing the term “bam file” in the description. The main section of the front page provides an overview on all available modules. Every module is visualized as a box that contains its name, author, assigned category and a short description. The search bar at the top can be used to filter modules using a keyword search, which can be applied to title, author, category and/or description. Alternatively, the modules displayed in the overview section can be filtered based

on authorship, category and update date. Clicking on a module box opens a detailed view, showing module dependencies, parameters and valid input values, return values and if applicable citation information and weblinks (see Fig. 3 for an example).

Public repositories for module and workflow sharing

Watchdog 2.0 now provides two repositories on Github under the *watchdog-wms* organization (`https://github.com/watchdog-wms/`) that are dedicated for sharing modules (`watchdog-wms-modules`) and workflows (`watchdog-wms-workflows`), respectively, by other users. In order to contribute either a module or workflow to one of the repositories, users have to first create a copy (fork) of the repository, change or add modules/workflows, commit the proposed changes to the repository copy, and submit these changes for review to the original repository via a pull request. An integration pipeline then checks whether the proposed changes adhere to essential requirements. If all automatic tests were successful, the proposed changes can be accepted by *Watchdog* team members.

Currently, the module repository contains 60 modules. Each module is located in a separate directory and must contain at least the XSD module file and an XML documentation file. Currently, most available modules focus on sequencing data analysis, in particular RNA-seq and ChIP-seq analysis. Some modules provide basic functionalities like file compression or text search while others fulfill more specific tasks, e.g. differential gene expression analysis (module *DETest*), peak detection in ChIP-seq data (module *GEM*) or identification of circular RNAs (modules *circRNAfinder* and *ciri2*). By default, modules are licensed under Apache License 2.0, but a different license can be assigned to a module by including it in the module folder. A reference book for all modules in the repository is available at `https://watchdog-wms.github.io/watchdog-wms-modules/`. It is automatically updated with every commit to the master branch of the module repository.

Workflows shared in the *watchdog-wms-workflows* repository also have to be located in separate directories. Each workflow directory has to contain the XML workflow file, a readme file and optionally example data. Workflows should be documented with inline comments. Furthermore, lines that require modifications to adapt e.g. to different computing environments or input data should be highlighted in order to allow everyone to quickly adapt the workflow. We recommend, but do not enforce, that paths or constant parameter values are not hard-coded in the task section of the workflow, but rather that global constants are defined in the settings section. A constant `CONSTANT` can then be referenced as `${CONSTANT}` within process block or task definitions. If this recommendation is followed, the workflow can be quickly adapted to a new environment or data by modifying only constants and executors.

Currently, the workflow repository contains e.g. the workflow for RNA-seq mapping and differential gene expression analysis from the original *Watchdog* release. Additionally, new workflows are available e.g. for circular RNA detection with CIRI2 [17] and circRNA_finder [18], ChIP-seq analysis using GEM followed by ChIPseeker [19, 20], and download of public NGS data from the NCBI Sequence Read Archive (SRA) [21] followed by alignment with HISAT2 [22].

Methods for ensuring reproducibility

A critical aspect of any analysis of biological data is the reproducibility of the results. While the use of a WMS already contributes to reproducibility, workflows may be modified between different runs of the workflow, e.g. by changing parameter values or in- or excluding some steps, or the underlying software may be changed, e.g. by updates to a new version. This may lead to uncertainty regarding the steps, parameters and software environment of the analysis that produced specific results. Furthermore, when reporting the individual steps of an analysis, for instance in a publication, some steps may be unintentionally omitted, making it difficult for others to reproduce the results. To address these problems, *Watchdog* 2.0 includes a number of new developments to ensure reproducibility of analyses.

Logging and automated reporting

When executing a workflow, *Watchdog* 2.0 now produces a time-stamped log file (filename extension `.resume`) reporting on the successful execution of each individual task. This log file is also used for the resume mode (see below). If a task creates multiple subtasks,

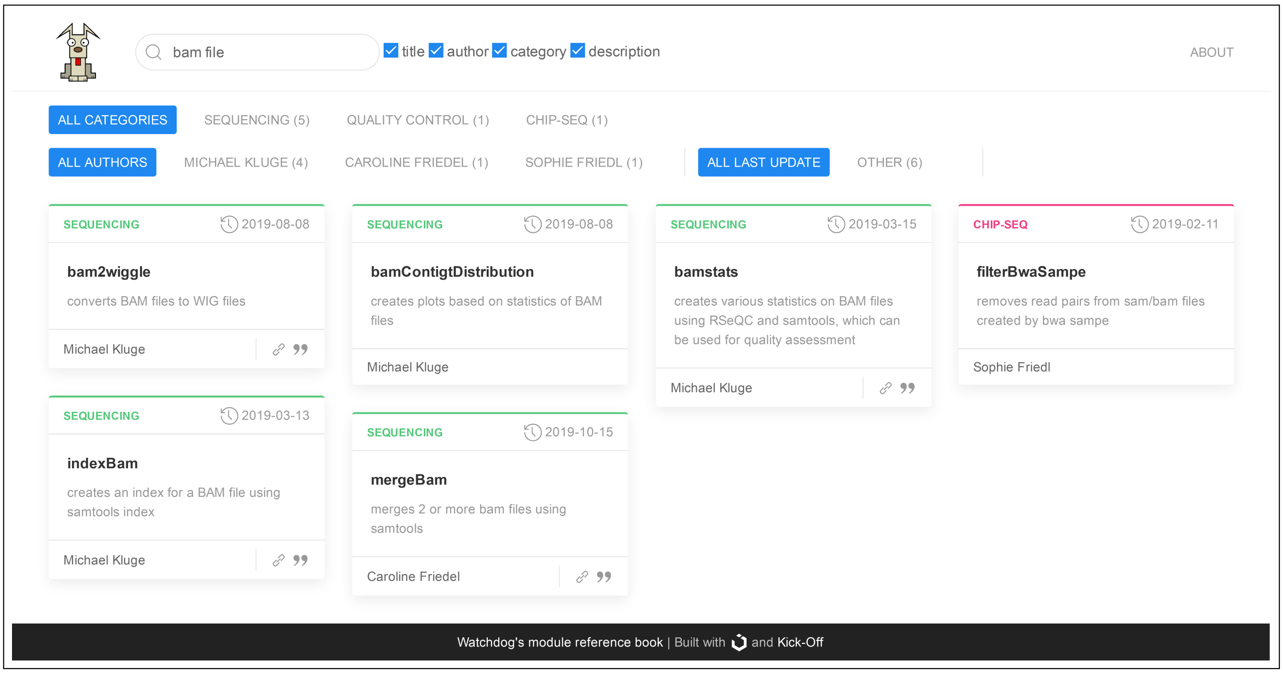

Figure 2. Overview page of the module reference book. The main section displays available modules as boxes, showing the module name, date of last change, a short description, links and the author of the module. A search bar and category bar can be used to filter the displayed modules using text search or multi-category filters. In this example, all modules containing the term “bam file” in the description are shown.

e.g. for multiple input samples, successful execution of each subtask is recorded. For each task/subtask the log file records the value of each input parameter as well as return values.

Moreover, a report of the executed steps can be automatically created from the log file using the new command-line tool *reportGenerator* provided with *Watchdog* 2.0 (see Fig. 4 for an example of the report). For this purpose, the XML documentation file of each module contains the element *paperDescription* which can be filled with a short description of the module and citation information. It can also contain references to parameters of the task or the software version (see below for software version logging). The *reportGenerator* concatenates these descriptions in the order the corresponding tasks were executed and replaces references by the values reported in the log file. There is also an option to include Pubmed IDs from the module documentation. The resulting report can then be used as a step-by-step protocol of the analysis or be further revised for the methods section of a manuscript.

Module versioning

Modules generally rely on third-party software that can be modified repeatedly to improve performance, fix bugs or be adapted to changing requirements, for instance by adding support for new types of experimental data. As a consequence, a module will need to be adapted over time, e.g. by changing the parameters of the module to support new parameters or drop obsolete ones. At the same, backward compatibility needs to be ensured such that previously defined workflows relying on the old module version can still be executed. One solution to this problem would be to duplicate the module and adapt the copy. However, this leads to unnecessary code duplication, as most of the module XSD file will remain unchanged, and results in code that is difficult to maintain.

To avoid this problem, *Watchdog* 2.0 now allows defining different versions of a module within one module XSD file by specifying the minimum and maximum supported module version for each element in the XSD file. If neither minimum or maximum supported version is indicated, the element is valid for all module versions. This allows changing input parameters, return values, or even the executed program call between different module versions. When executing a workflow, the module version for each task will also be recorded in the log file. By default, the first version of a module is used unless otherwise specified in the workflow XML file. This guarantees that workflows defined before a new module version was introduced do not have to be adapted.

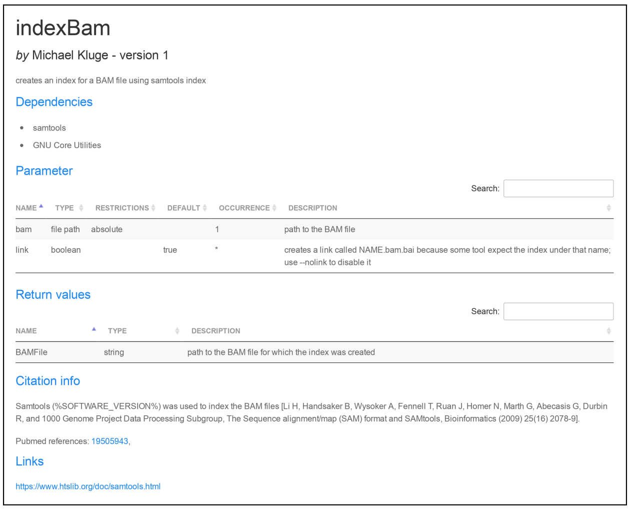

Figure 3. Detailed view of a module in the reference book. As an example, the detailed view on the *indexBam* module is shown, containing a short description, dependencies on third-party software, parameters with valid ranges and descriptions, return values, citation information and weblinks. The citation information will also be included into the step-by-step report automatically created from the workflow execution log file.

> Quality of the sequencing data was checked using FastQC (0.11.3). RNA-seq reads were mapped against the XXX genome using ContextMap (2.7.9) with BWA as short read aligner and default parameters. Samtools (1.9) was used to convert SAM to BAM files. Samtools (1.9) was used to index the BAM files. Quality of the resulting mappings was assessed using RSeQC (3.0.0). FeatureCounts (1.4.6) was applied to count read/fragment counts per gene/exon/other feature according to Ensembl_Homo_sapiens.GRCh38.78.chr21.gtf annotation. Differential gene expression analysis was performed using DESeq2 (1.22.2).

Figure 4. Result of automated report generation for example workflow. This example shows the step-by-step analysis report generated with the *reportGenerator* from the execution log file for the RNA-seq example workflow provided with *Watchdog*. The workflow was described in detail in our original *Watchdog* publication [12]. The annotation file name (a parameter to featureCounts) and software version numbers in brackets are automatically obtained from the log file (see software version logging). For this example, the workflow was simplified to perform differential gene expression analysis only with DESeq2, instead of four different gene expression analysis methods as previously described. For modules without paper description (e.g. unzipping or replicate merging), the report would contain the text "No short description given in documentation of module *module name*". To shorten this example, these sentences were manually removed as well as the citation information commonly included in the module descriptions.

Software version logging

Watchdog is very flexible with regard to how dependencies to third-party software in a module can be handled by module developers. Software can be shipped with the module, loaded via package and environment management systems like Conda [13] or be required to be installed on the system that will execute the corresponding task (e.g. the local host or a computer cluster). In any case, it is crucial to know which software versions were run for a particular analysis in order to reproduce the analysis results or understand differences in outputs between repeated runs since new software releases often correct errors or may change the behavior of the software.

Thus, *Watchdog* 2.0 now implements a general approach for reporting versions of third-party software used in a module in the log file. For this purpose, a new attribute in the module XSD file can be used to define the flag for version printing of third-party software. During workflow execution, after a task or subtask has been completed successfully on a particular computer, the program call defined in the corresponding module is invoked with the version flag on the same computer to retrieve the installed third-party software version. This software version is then reported for the task/subtask in the log file. If the version flag has not been defined in the module, this step is omitted for the corresponding tasks. This option is also useful for identifying differences in installed third-party software between different executors used for workflow execution, such as the local host, a computer cluster or remote executors accessed by SSH.

Execution wrappers

A disadvantage of *Watchdog*'s flexibility on how installation of third-party software is handled is that it complicates both reusability and reproducibility of workflows. Having to install all required software before modules or workflows can be used can be cumbersome. Furthermore, to fully reproduce results from a workflow, users would have to make sure that they (still) have the same software versions installed as in the original run of a workflow. Thus, we now implemented execution wrappers to explicit support automatic deployment of software via package managers or container virtualization in *Watchdog* 2.0. Execution wrappers are initialized in the settings section of a Watchdog workflow and are then assigned to individual executors, which in turn use the wrapper to deploy the software for all

tasks they run. Each executor can be assigned both a package manager and a container, thus package managers can also be used within containers. Furthermore, different packager managers or containers can be assigned to different tasks by using different executors and corresponding execution wrappers for these tasks. Execution wrappers are implemented using *Watchdog*'s plugin system, thus the set of available execution wrappers can be extended by users without having to modify the *Watchdog* code.

Currently, *Watchdog* 2.0 provides execution wrappers for the Conda package manager [13] and for Docker container virtualization [14]. To enable use of Conda for a module, the module directory only has to contain a YAML file defining the default Conda environment (`modulename.conda.yml`). For different versions of a module, different Conda environments can be defined (ending in `.v[0-9]+.conda.yml`). If no version-specific Conda definition file is found, the default Conda environment for the module is used. If Conda execution wrappers are not employed in a workflow or for a particular executor, the Conda environment definition will simply be ignored for the whole workflow or the tasks run by the executor, respectively. Thus, previously developed workflows will not be affected by these changes.

The Docker execution wrapper allows running tasks within containers built from Docker images using Docker, Podman or Singularity. Furthermore, it provides an option for automatically mounting files and directories on the host machine that are used in parameters of tasks. This option is enabled by default but can be disabled. Thus, adding container virtualization to an executor does not require changes to corresponding tasks. An example for using Docker and Conda in combination is provided in the workflow for RNA-seq mapping and differential gene expression analysis available from the workflow repository and with the *Watchdog* distribution.

New execution modes

In the original *Watchdog* version, the *Watchdog* scheduler had to run continuously on the computer on which workflow execution is started. If workflow execution was interrupted, e.g. by a computer crash or reboot, only a manual restart option was available. This required identifying the last task finished successfully or re-running some analyses in case only some subtasks of a task finished successfully. To avoid this problem, *Watchdog* 2.0 now supports two additional execution modes (see Fig. 5). The first one allows resuming workflow execution at any point and re-running only the tasks or subtasks in a workflow that did not finish successfully, were modified or depended on modified tasks. The second execution mode allows detaching from workflow execution by shutting down the *Watchdog* scheduler on the current computer while tasks distributed to a computer cluster continue running. The scheduler can then reattach to the workflow execution at a later time either from the same or a different computer. This can be used for instance to reboot the machine running the scheduler or to switch from a desktop computer to a laptop without interrupting execution of tasks running on a computer cluster.

Resume mode

As described above, *Watchdog* 2.0 creates a detailed log file during execution of a workflow containing successfully finished (sub)tasks as well as their input parameters and return values. In resume mode, *Watchdog* 2.0 uses the log file of a previous workflow run to determine which (sub)tasks have to be (re-)executed or not. Individual (sub)tasks are identified by their input parameter combinations. (Sub)tasks not listed in the log file with exactly the same input parameter values will be scheduled to be executed. Furthermore, (sub)tasks that previously finished successfully with the same parameters are re-executed if they depend on other (sub)tasks that are (re-)run.

This not only allows resuming workflows that were interrupted

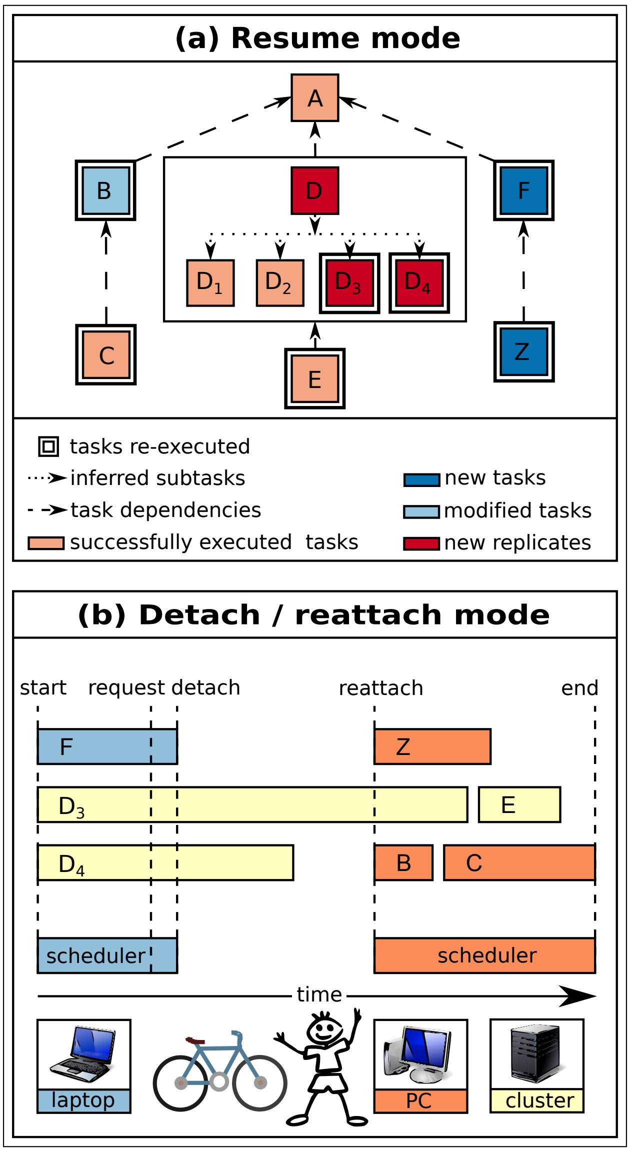

Figure 5. New execution modes in *Watchdog* 2.0. (a) Resume mode: From the log file of a previous workflow execution and the workflow XML file, the *Watchdog* scheduler automatically detects (sub)tasks that either have not yet run successfully, are new or modified or require processing of additional samples. Consequently, only these (sub)tasks are executed as well as all (sub)tasks depending on them (dependencies indicated as dashed lines in this figure). Here, light red indicates (sub)tasks that were previously executed successfully, light blue tasks that were since modified, dark blue new tasks that were added and dark red additional subtasks that have to be executed because additional samples were added. Double lines around tasks indicate which (sub)tasks have to be (re-)executed after resuming this workflow. **(b)** Visualization of the detach/reattach mode after resuming the workflow shown in (a). In this case, subtasks of *D* and task *E* are executed on a computer cluster, while a local executor is used for all other tasks. In this example, the *Watchdog* scheduler is originally started on a laptop and some tasks are scheduled and executed. After a while, a detach request is sent and no more new tasks are scheduled on the local host. Once the tasks on the local computer (blue) have finished, the detach file is written and the scheduler terminates. Subtasks D_3 and D_4 submitted to the computer cluster (yellow) continue to be executed. When the user reattaches to workflow execution, this time on a desktop computer (orange), new tasks are again scheduled.

unexpectedly (e.g. by hardware failure or power outage) but also workflows that were modified, i.e. by changing parameters for some tasks, without unnecessarily re-running tasks. Here, *Watchdog* 2.0 guarantees that all results are updated that may be affected by the modification. Furthermore, additional samples, e.g. for other condi-

tions or more replicates, can be easily included without re-running analyses for samples already processed. Importantly, identification of (sub)tasks that require (re-)execution is performed automatically without manual user input. This reduces both the overhead for the user and eliminates the risk that they may forget some steps that need to be repeated.

The *Watchdog* 2.0 resume mode is illustrated in Fig. 5(a) for an example workflow. In this case, a task was modified (task B), additional samples were added to task D (marked red), requiring additional subtasks to be run, and additional tasks were added (F and Z). In resume mode, task B will be re-run because of modified parameters and task C because it depends on task B. For task D, only the new subtasks will be executed, but task E will be repeated as it depends on D. In addition, the newly added tasks will be run.

Detach / reattach mode

In most cases, the *Watchdog* scheduler will run on a laptop or desktop computer and outsource all resource-intensive tasks to a distributed computer system, e.g. a computer cluster. As execution of long, resource-intensive workflows may take hours or even days to complete, it may not always be possible for the *Watchdog* scheduler to be running continuously on the host computer. For instance, the host running *Watchdog* might require a reboot to install software updates or dedicated computer cluster submission hosts may not allow long-running programs. If the *Watchdog* scheduler is run on a laptop, the user may want to change locations with their laptop. To support these use cases, *Watchdog* 2.0 now provides the option to detach the scheduler from a running workflow and reattach at a later time. Notably, the user does not have to decide before execution whether to use this mode, but can decide to detach at any time after starting execution in either normal or resume mode.

In *Watchdog* 2.0, the user can request to detach using a keystroke combination (Ctrl-C) or a link in the email notification. After the request is sent, *Watchdog* will wait for tasks to complete that are running either on the local host or a remote host via SSH, but schedule no further tasks on these executors. In contrast, *Watchdog* will continue to submit tasks on cluster executors with workload managers working independently of *Watchdog* (currently SGE and SLURM are supported). Once all tasks on the local and remote hosts are finished, *Watchdog* will save the information on tasks running on cluster executors to a file and then terminate itself. From this moment, tasks already running on or submitted to computing clusters will continue running or be scheduled to run by the corresponding workload managers, but no new tasks can be submitted to these clusters.

The detach file can then be used at a later time to reattach to workflow execution at the point where it was stopped previously. *Watchdog* will then obtain information on the execution status of tasks that were still running on or submitted to computer clusters before detaching, i.e. whether they are still running or finished successfully or with errors, and continue scheduling tasks on all executors accordingly. Notably, the *Watchdog* scheduler can also be reattached on another computer using the detach file, allowing for instance to switch from a laptop at home to a desktop computer at work as illustrated in Fig. 5(b). Moreover, *Watchdog* 2.0 also provides a command-line tool to periodically start the scheduler in auto-detach mode. In this mode, the scheduler checks if tasks were finished successfully, submits new tasks if possible and then terminates itself automatically.

Comparison to other WMSs

In this article, we present a number of new developments in our WMS *Watchdog*. The previously published version of *Watchdog* [12] was already extensively compared against the most popular WMSs for biological analyses, i.e. *Galaxy* [8], *KNIME* [9], *Snakemake* [10], and *Nextflow* [11] (see Fig. 12 in [12] for this comparison). Compared features included e.g. availability of GUIs/web interfaces for workflow design, execution and monitoring, support for parallel, distributed and cloud computing, dependency definition and many more. This showed that *Watchdog* combined features of existing WMSs and provided novel useful features for execution and monitoring of workflows both for users with and without programming skills.

Since these features are essentially unchanged, we will not repeat this comparison here, but refer to our original publication [12]. In the following, we will discuss how *Watchdog* 2.0 compares to these other WMSs regarding the new features we present in this article since these were not previously analyzed. First, we provide a very brief description of *Galaxy*, *KNIME*, *Snakemake* and *Nextflow*. For more details, please refer to our original publication [12].

Galaxy is targeted at experimentalists without programming experience and allows performing data analyses in the web browser. Workflows can be constructed on public or private *Galaxy* servers in a web-based user interface from a set of available tools and can then be executed. New tools for use in a *Galaxy* workflow are defined in an XML format specifying the input parameters for this tool as well as the program to execute.

KNIME is an open-source data analysis platform based on the Eclipse integrated development environment (IDE). It provides a powerful GUI for workflow construction, execution and visualization of results, which can also be used without programming experience. Java programming skills are required for making a new tool available in a so-called node as multiple Java classes have to be extended.

Snakemake uses a Python-based language to define workflows in a so-called Snakefile as a set of rules that describe how output files are created from input files. Dependencies between rules are determined automatically based on input and output files and the order of rule execution is determined upon invocation based on these dependencies. Encapsulation of re-usable components can be performed using so-called wrappers. Writing workflows and wrappers requires knowledge of the *Snakemake* syntax and some degree of programming skills.

Nextflow extends the Unix pipes model to transfer complex data between consecutive processes as shared data streams. It provides its own scripting language based on the Groovy programming language to define workflows. Individual analysis steps are defined as processes in the *Nextflow* workflow itself, thus no actual encapsulation of tools into re-usable components is supported. Similar to *Snakemake*, programming experience is required to define workflows and no GUI is provided.

For the following comparison, features were grouped broadly into categories reusability, reproducibility and workflow execution. A summary of the comparison is shown in Table 1.

Reusability

For this part of the comparison, we focused on features that support development and sharing of tools (modules in *Watchdog*, tools in *Galaxy*, nodes in *KNIME*, rules in *Snakemake*, processes in *Nextflow*) for (re-)use in multiple analysis workflows as well as sharing and repurposing of existing workflows (F1-F7 in Table 1). As there is no real encapsulation of tools in *Nextflow*, most of these features are not applicable to it.

Support for tool creation (F1) is provided in *Galaxy* by the command-line program Planemo, which is similar to the helper script originally provided by *Watchdog* for module creation. Notably, Planemo also requires manually adding all parameters for a new tool. For *KNIME*, an Eclipse extension (KNIME Node Wizard) is available, which generates the project structure, the plug-in manifest and all required Java classes. However, the Java classes only contain the basic backbone (in particular no parameters or flags) and have to be massively extended by the developer. *Snakemake* does not provide any software or script for defining wrappers.

All three WMSs allow documenting (F2) tools and their parameters in XML or YAML format. In case of *Snakemake*, the specifica-

tion does not require to explicitly document parameters and in- and output. Instead, an example Snakefile showing the use of the wrapper has to be provided. A reference book containing information on all available tools (F3) can be generated for *Snakemake* wrappers as a separate webpage. This contains the example Snakefile, the code of the wrapper, author information and software dependencies. In contrast, the documentation of *KNIME* nodes and *Galaxy* tools, respectively, is displayed on their respective GUI/web interface during workflow creation. Furthermore, all three WMSs perform tool versioning (F4).

For sharing tools (F5) or complete workflows (F6) with other users, *Galaxy* and *KNIME* operate dedicated sharing platforms [23, 24], while *Snakemake* provides source code repositories similar to *Watchdog* 2.0 [25, 26]. Furthermore, dedicated sharing platforms are operated by the *KNIME* and *Nextflow* community [27, 28].

Repurposing an existing workflow for new data (F7) requires different steps in the different WMSs. In *Galaxy* and *KNIME*, existing workflows can be imported and subsequently input files or values have to be selected/modified in the web interface and GUI, respectively. For *Nextflow*, input is provided via command-line parameters. For *Snakemake*, relative paths to input files are hard-coded in the Snakefile. Thus, repurposing a *Snakemake* workflow only requires copying the Snakefile to a directory in which input files are stored or linked in the subdirectory structure used in the Snakefile. In *Watchdog* workflows, input files and parameters are also hard-coded but absolute paths are used. In a well-designed workflow, global constants are defined for input values and files in the settings section and used throughout the workflow. Thus, repurposing only requires editing these constants either in a text or XML editor or the GUI. This is not more effort than required by other WMSs, with the exception of *Snakemake*. However, it provides more flexibility than *Snakemake* regarding how input data is distributed in the file system and workflows can be stored anywhere, e.g. in a directory containing all previously developed workflows.

Reproducibility

Here, we focus on features (F8-F11) related to reproducibility of analysis results carried out at an earlier time, on different computer systems and/or by other scientists. Most of the other WMSs do not support explicit logging of external software during workflow execution similar to *Watchdog* 2.0 (F8). However, *Galaxy*, *Snakemake* and *Nextflow* support controlling external software dependencies and versions with the Conda package manager or using Docker containers (F9). Furthermore, *Snakemake* reports on executed workflows (see next paragraph) display the Conda environment for each task, including software versions.

A description of all performed analysis steps (F10) can be obtained in *Snakemake* and *Nextflow* through generation of HTML reports, in which individual steps are listed in a table format and in case of *Snakemake* also visualized as a graph. *Galaxy* displays all executed tasks as a list in its analysis history. In contrast, *KNIME* supports only static workflow descriptions that have to be prepared by the workflow developer. The dynamic report created by *Watchdog* 2.0 from the execution log does not only list performed steps, but includes short descriptions of each step prepared by module developers with citation information and (optionally) Pubmed IDs (F11). The only other WMS allowing to declare citations for tools is *Galaxy*. In this case, a list containing citations for all used tools can be exported after executing a workflow in *Galaxy*. None of the other WMSs support creation of a step-by-step report for inclusion in a manuscript draft similar to *Watchdog* 2.0.

Execution

All WMSs except *Galaxy* can resume execution of partly executed workflows (F12) and are able to detect new tasks, modified tasks or tasks with altered dependencies and consequently execute only these tasks (F13). With *Snakemake* and *Nextflow*, new samples (e.g. additional replicates) can be included in an analysis workflow without having to reprocess all samples (F14), but this option has to be forcibly triggered in *Snakemake*. This is not possible for *KNIME* workflows. One possibility to avoid unnecessary reprocessing in *KNIME* is to implement *KNIME* nodes that can detect if the corresponding task was already executed successfully on a sample as done by Hastreiter *et al.* [29]. However, this adds additional overhead for node development.

Finally, similar execution modes to the detach/reattach mode of *Watchdog* 2.0 (F15) are at least partly supported by all compared WMSs apart from *Nextflow*. Since *Galaxy* is a web-based system, the user can log off (detach) and log in (reattach) at any time and from different client systems. Furthermore, the *Galaxy* server can also be restarted while tasks continue running on a computer cluster if no tasks are executed locally on the server. In *KNIME*, remote execution is only possible with non-free extensions like the KNIME Server or a cluster extension. If tasks are executed remotely using such an extension, the local *KNIME* instance can be detached and reattached to workflow execution. Finally, *Snakemake* provides the option to stop scheduling by sending the TERM signal and wait for all jobs to be finished before terminating. Later, workflow execution can then simply be resumed. However, this mode also stops scheduling of jobs on computer clusters and waits for jobs running on computer clusters to be finished. Alternatively, Ctrl+C kills the main Snakemake process and all jobs running on the local computer, but jobs already running on a computing cluster keep running. With the correct use of profiles, it is then possible for the workflow to check the status of those jobs after a restart.

Conclusion

In this article, we present the new developments in *Watchdog* 2.0, which focus on improving reusability of modules and workflows, reproducibility of analysis results and convenience of workflow execution.

To simplify module development, we developed the *moduleMaker* GUI for semi-automatically creating a module for a software by parsing its help page. Manual overhead for the module creator is then mostly limited to choosing the best regular expression set, validating and correcting automatically identified parameters and adding additional parameters or return values considered necessary. Furthermore, we established public sharing repositories to support and encourage exchange of developed modules and workflows between scientists. Modules are now documented in a standardized documentation format, from which an HTML-based module reference book can automatically be created. The reference book provides an overview and details on available modules and can be easily regenerated to integrate new modules, e.g. modules created by other developers.

To guarantee reproducibility of workflow results, we introduced module versions and extensive logging of successfully executed steps including parameter values and third-party software versions. From the log file of a workflow execution, a report can then be automatically generated that serves both as a documentation of the analysis steps and as a starting point for drafting the corresponding methods section of a manuscript. This not only reduces the effort in creating a description of the analysis, it also prevents accidental omission of individual steps. In addition, *Watchdog* 2.0 now provides integrated support for automatic deployment of software, in particular with Conda or Docker, in the form of execution wrappers.

Finally, with the new resume and detach/reattach execution mode, convenience and flexibility of workflow execution is greatly enhanced in *Watchdog* 2.0. The resume mode not only implements the state-of-the-art for WMSs that allows resuming interrupted workflow execution, but automatically identifies and re-executes tasks with modified parameters or additional input samples as well as downstream tasks that depend on them. The detach/reattach mode allows shutting down the *Watchdog* scheduler on a local computer

		Feature	*Watchdog*	*Galaxy*	*KNIME*	*Snakemake*	*Nextflow*
reusability	F1	Support for tool creation	command-line/GUI	command-line[1]	Eclipse Wizard	no	n.a.
	F2	Tool documentation	XML based	XML based	XML based	YAML based[2]	n.a.
	F3	Tool reference book	webpage generator	part of GUI	part of GUI	webpage generator	n.a.
	F4	Tool versioning	yes	yes	yes	yes	n.a.
	F5	Sharing of tools	repository[3]	ToolShed[4]	KNIME Hub[5] / NodePit[6,*]	repository[7]	n.a.
	F6	Sharing of workflows				repository[8]	nf-core[9,*]
	F7	Repurposing of workflows	XML edit/GUI	GUI	GUI	copy Snakefile	command-line
reproducibility	F8	Software version logging	yes	no	no	yes	no
	F9	Software deployment	execution wrappers/ Conda, Docker	Conda, Docker	no	Conda, Docker	Conda, Docker
	F10	Creation of workflow report	yes	list via history	static description[10]	HTML report	HTML report
	F11	Citation export	yes	yes	no	no	no
execution	F12	Resume workflow	yes	no	yes	yes	yes
	F13	Process only updated tasks	yes	no	yes	yes[11]	yes
	F14	Process only new replicates	yes	no	no	yes[11]	yes
	F15	Detach / reattach	yes	yes[12]	non-free feature[13]	yes[14]	no

Table 1. Comparison of *Watchdog* with four other commonly used WMSs. The selected WMSs are compared against *Watchdog* based on features grouped broadly into the categories reusability, reproducibility, and execution. Footnotes: [1] Python-based command-line program (Planemo); [2] no explicit documentation of parameters but example Snakefile and wrapper source code is part of the documentation; [3] https://github.com/watchdog-wms; [4] [23]; [5] [24]; [6] [27]; [7] [25]; [8] [26]; [9] [28]; [10] a description that was manually created for a specific workflow can be displayed but is not dynamically created; [11] flag `--list-params-changes` or `--list-input-changes` in combination with the `--forcerun` flag; [12] client: anytime / server: if jobs are not executed locally on the server; [13] non-free SGE extension or KNIME server required; [14] sending of a TERM signal stops scheduling of new jobs and waits for all running jobs to finish; Ctrl+C kills all jobs running on the local computer, jobs running on a computing cluster continue to run; [*] community project

while jobs continue to be executed on computer clusters. The user can then reattach to workflow execution and resume scheduling of tasks at a later time and even from a different computer.

While many of the new features in *Watchdog* 2.0 are also present in other popular WMSs, none are implemented in all of them. Furthermore, even if these features are available in other WMSs, the implementations in *Watchdog* 2.0 often add additional capabilities, such as e.g. the possibility to automatically generate a step-by-step report. Combined with the existing advantages of *Watchdog* highlighted in our original publication, we thus believe that *Watchdog* 2.0 will be of great benefit to users with a wide range of computer skills for performing large-scale bioinformatics analyses in a flexible and reproducible manner.

Availability of source code and requirements

- Project name: Watchdog 2.0
- Project home page: https://www.bio.ifi.lmu.de/watchdog
- Source code: https://github.com/klugem/watchdog, https://github.com/watchdog-wms
- Operating system(s): Platform independent
- Programming language: Java
- Other requirements: Java 11 or higher, JavaFX 11 or higher for the GUIs, individual requirements for modules
- License: GNU General Public License v3.0
- DOI: https://doi.org/10.5281/zenodo.3764538
- RRID: SCR_018355
- biotoolsID: biotools:watchdog

Declarations

List of abbreviations

GUI graphical user interface
IDE integrated development environment
NGS next-generation-sequencing
WMS workflow management system

Competing Interests

The authors declare that they have no competing interests.

Funding

This work was supported by grants FR2938/7-1, FR2938/10-1 and CRC 1123 (Z2) from the Deutsche Forschungsgemeinschaft (DFG) to CCF.

Author's Contributions

MK developed the software and wrote the manuscript. M-SF tested *Watchdog* 2.0 and implemented modules and the workflow for the analysis of circular RNAs in high-throughput sequencing data. ALM implemented the *moduleMaker* GUI under supervision of CCF and MK. CCF tested the software, helped in revising the manuscript and supervised the project. All authors read and approved the final manuscript.

References

1. Hayden EC. Technology: The $1,000 genome. Nature 2014;507:294–295.
2. Consortium IHGS. Finishing the euchromatic sequence of the human genome. Nature 2004;431:931–945.
3. Wang Z, Gerstein M, Snyder M. RNA-Seq: a revolutionary tool for transcriptomics. Nature reviews Genetics 2009;10:57–63.
4. Furey TS. ChIP-seq and beyond: new and improved methodologies to detect and characterize protein-DNA interactions. Nature reviews Genetics 2012;13:840–852.
5. Buenrostro JD, Wu B, Chang HY, Greenleaf WJ. ATAC-seq:

A Method for Assaying Chromatin Accessibility Genome-Wide. Current protocols in molecular biology 2015;109:21.29.1–9.
6. Guo W, Tzioutziou N, Stephen G, Milne I, Calixto C, Waugh R, et al. 3D RNA-seq - a powerful and flexible tool for rapid and accurate differential expression and alternative splicing analysis of RNA-seq data for biologists. bioRxiv 2019;.
7. Sundararajan Z, Knoll R, Hombach P, Becker M, Schultze JL, Ulas T. Shiny-Seq: advanced guided transcriptome analysis. BMC Research Notes 2019;12:432.
8. Taylor J, Schenck I, Blankenberg D, Nekrutenko A. Using galaxy to perform large-scale interactive data analyses. Current protocols in bioinformatics 2007;Chapter 10:Unit 10.5.
9. Berthold MR, Cebron N, Dill F, Gabriel TR, Kötter T, Meinl T, et al. KNIME: The Konstanz Information Miner. In: Studies in Classification, Data Analysis, and Knowledge Organization (GfKL 2007) Heidelberg-Berlin: Springer; 2007. p. 319–26.
10. Köster J, Rahmann S. Snakemake–a scalable bioinformatics workflow engine. Bioinformatics 2012;28:2520–2.
11. Di Tommaso P, Chatzou M, Floden EW, Barja PP, Palumbo E, Notredame C. Nextflow enables reproducible computational workflows. Nature biotechnology 2017;35:316–9.
12. Kluge M, Friedel CC. Watchdog – a workflow management system for the distributed analysis of large-scale experimental data. BMC Bioinformatics 2018;19:97.
13. Conda. https://conda.io, accessed 11 Nov 2019.
14. Docker. https://www.docker.com/, accessed 01 Apr 2020.
15. McAffer J, Lemieux JM, Aniszczyk C. Eclipse Rich Client Platform. 2nd ed. Boston, US: Addison-Wesley Professional; 2010.
16. UIkit. https://getuikit.com, accessed 11 Nov 2019.
17. Gao Y, Zhang J, Zhao F. Circular RNA identification based on multiple seed matching. Briefings in bioinformatics 2018;19:803–810.
18. Westholm JO, Miura P, Olson S, Shenker S, Joseph B, Sanfilippo P, et al. Genome-wide analysis of drosophila circular RNAs reveals their structural and sequence properties and age-dependent neural accumulation. Cell reports 2014;9:1966–1980.
19. Guo Y, Mahony S, Gifford DK. High resolution genome wide binding event finding and motif discovery reveals transcription factor spatial binding constraints. PLoS computational biology 2012;8(8):e1002638.
20. Yu G, Wang LG, He QY. ChIPseeker: an R/Bioconductor package for ChIP peak annotation, comparison and visualization. Bioinformatics (Oxford, England) 2015;31(14):2382–2383.
21. Leinonen R, Sugawara H, Shumway M, Collaboration INSD. The sequence read archive. Nucleic acids research 2011;39:D19–D21.
22. Kim D, Paggi JM, Park C, Bennett C, Salzberg SL. Graph-based genome alignment and genotyping with HISAT2 and HISAT-genotype. Nature biotechnology 2019;37:907–915.
23. Galaxy Tool Shed. https://toolshed.g2.bx.psu.edu, accessed 11 Nov 2019.
24. KNIME Hub. https://hub.knime.com, accessed 11 Nov 2019.
25. SnakeMake Wrappers repository. https://bitbucket.org/snakemake/snakemake-wrappers, accessed 11 Nov 2019.
26. SnakeMake Workflows repository. https://github.com/snakemake-workflows, accessed 11 Nov 2019.
27. NodePit. https://nodepit.com, accessed 11 Nov 2019.
28. nf-core. https://nf-co.re, accessed 11 Nov 2019.
29. Hastreiter M, Jeske T, Hoser J, Kluge M, Ahomaa K, Friedl MS, et al. KNIME4NGS: a comprehensive toolbox for next generation sequencing analysis. Bioinformatics 2017;33:1565–1567.

Click here to access/download
Supplementary Material
Response_to_reviewers.pdf

Manuscript with changes marked

Click here to access/download
Supplementary Material
main_marked.pdf
