# Supplementary material for: Watchdog 2.0: New developments for reusability, reproducibility, and workflow execution
Source: Gigascience. 2020 Jun 17;9(6):giaa068. doi: 10.1093/gigascience/giaa068 (PMC7298769; doi:10.1093/gigascience/giaa068)
Supplement: giaa068_Response_to_Reviewer_Comments_Original_Submission [file giaa068_response_to_reviewer_comments_original_submission.pdf]

Author's Response To Reviewer Comments

Close

The response to reviewers was uploaded as a separate PDF as Supplementary Material to the submission.

Close
