# Supplementary material for: Watchdog 2.0: New developments for reusability, reproducibility, and workflow execution
Source: Gigascience. 2020 Jun 17;9(6):giaa068. doi: 10.1093/gigascience/giaa068 (PMC7298769; doi:10.1093/gigascience/giaa068)
Supplement: giaa068_Response_to_Reviewer_Comments_Revision_1 [file giaa068_response_to_reviewer_comments_revision_1.pdf]

Author's Response To Reviewer Comments

Close

The response to reviewer and editor comments was uploaded as a separate PDF as Supplementary Material to the submission.

Please note that the file created by the submission system from the uploaded LaTeX file does not show the citations, references and the list of abbreviations correctly. The main manuscript file main.pdf we uploaded as well shows these correctly.

Close
