# Supplementary material for: Watchdog 2.0: New developments for reusability, reproducibility, and workflow execution
Source: Gigascience. 2020 Jun 17;9(6):giaa068. doi: 10.1093/gigascience/giaa068 (PMC7298769; doi:10.1093/gigascience/giaa068)
Supplement: giaa068_Reviewer_1_Report_Original_Submission — Tazro Ohta -- 1/2/2020 Reviewed [file giaa068_reviewer_1_report_original_submission.pdf]

Reviewer Report

Title: Watchdog 2.0: New developments for reusability, reproducibility and workflow execution

Version: Original Submission **Date:** 1/2/2020

Reviewer name: Tazro Ohta

Reviewer Comments to Author:

In this manuscript, the authors described the Watchdog 2.0, a workflow management system implemented in Java. The system uses its own XML based syntax for a module (tool) and workflow description. The authors explained the features introduced in version 2.0 with the following three topics, reusability of modules, reproducibility of workflow execution, and workflow execution control. For the reusability of modules, the system offers a GUI helper tool for module design, the module documentation system, and the public GitHub repository to share modules among the Watchdog users. For the reproducibility of workflow execution, the system automatically generates a workflow execution report with module versions and software versions used in the modules. And for workflow execution control, two new execution mode was introduced; resume of workflow runs and detach/re-attach to the workflow runner. The authors described no other workflow management system is implemented with all of them, which makes the system unique. All these features are beneficial for most researchers who do genomic data analysis. The system working with all these features is also a good example for the other workflow system developers. The manuscript also has a fair comparison with existing workflow management systems which I can agree with (I am a user of Galaxy, Common Workflow Language, and nextflow, have no practical experience with KNIME and Snakemake).
This manuscript focuses on the explanation of the updates in version 2.0, thus it is fair to have it as a technical note. This is not an article that reports novel insights, so it would sound better to change the section name "Findings" to "Implementations" or similar. I have tested the Watchdog version 2.0.4 (1771r) on macOS 10.14.1 and confirmed that the GUI module creation tool WorkflowDesigner and the Watchdog worked with the features described in the manuscript. The manuscript is well written, so only minor comments for documentation and overall software design are below.
1. Installation of dependencies on the GitHub README
The system works with Java 11 and requires the installation of the JavaFX SDK. The Watchdog system is well designed also for non-programmer users, and the authors also claimed it in the manuscript, but it is not obvious for those users how to install Java version 11 and the JavaFX SDK. Please update the documentation on the GitHub repo to include enough guidance to install the dependencies on different platforms such as Windows, Mac, and Linux. It is also worth mentioning that there are the docker images on Docker Hub. I also recommend using markdown format for README so that the document has better readability on GitHub.
2. "Getting started" in README
This is just a recommendation for the project, but it would be very helpful for the beginners to have a guide to start designing their workflows and run on their computers first. I had to read the help message of the provided command-line tool and GUI menu to run my workflow, which may be a complicated

procedure for non-programmer users.
3. The license of shared modules
The module sharing repository with automated testing is very practical to ensure the quality of shared modules. However, the repository has the license file of GPL-3.0. Is it possible to assign a different license for each shared module and still in the repository if a user needed? if so, please indicate it in the manuscript.
4. Tool execution in Linux containers
It is slightly strange that there are no mentions of container virtualization such as Docker or Singularity when the authors explain many features to support the reproducibility of workflow execution such as tool version management. Is there any limitation that the system cannot support tool execution in a container? If so, please indicate it in the manuscript.
Tazro Ohta

Level of Interest

Please indicate how interesting you found the manuscript: Choose an item.

Quality of Written English

Please indicate the quality of language in the manuscript: Choose an item.

Declaration of Competing Interests

Please complete a declaration of competing interests, considering the following questions:

- Have you in the past five years received reimbursements, fees, funding, or salary from an organisation that may in any way gain or lose financially from the publication of this manuscript, either now or in the future?
- Do you hold any stocks or shares in an organisation that may in any way gain or lose financially from the publication of this manuscript, either now or in the future?
- Do you hold or are you currently applying for any patents relating to the content of the manuscript?
- Have you received reimbursements, fees, funding, or salary from an organization that holds or has applied for patents relating to the content of the manuscript?
- Do you have any other financial competing interests?
- Do you have any non-financial competing interests in relation to this paper?

If you can answer no to all of the above, write 'I declare that I have no competing interests' below. If your reply is yes to any, please give details below.

I declare that I have no competing interests

I agree to the open peer review policy of the journal. I understand that my name will be included on my report to the authors and, if the manuscript is accepted for publication, my named report including any attachments I upload will be posted on the website along with the authors' responses. I agree for my report to be made available under an Open Access Creative Commons CC-BY license (http://creativecommons.org/licenses/by/4.0/). I understand that any comments which I do not wish to be included in my named report can be included as confidential comments to the editors, which will not be published.

Choose an item.

To further support our reviewers, we have joined with Publons, where you can gain additional credit to further highlight your hard work (see: https://publons.com/journal/530/gigascience). On publication of this paper, your review will be automatically added to Publons, you can then choose whether or not to claim your Publons credit. I understand this statement.

Yes Choose an item.
