# Supplementary material for: Watchdog 2.0: New developments for reusability, reproducibility, and workflow execution
Source: Gigascience. 2020 Jun 17;9(6):giaa068. doi: 10.1093/gigascience/giaa068 (PMC7298769; doi:10.1093/gigascience/giaa068)
Supplement: giaa068_Reviewer_2_Report_Original_Submission — Jan Forster -- 1/31/2020 Reviewed [file giaa068_reviewer_2_report_original_submission.pdf]

Reviewer Report

Title: Watchdog 2.0: New developments for reusability, reproducibility and workflow execution

Version: Original Submission **Date:** 1/31/2020

Reviewer name: Jan Forster

Reviewer Comments to Author:

The authors present Watchdog 2.0, an updated version of their WMS watchdog.
New additions presented in the paper include platforms for sharing modules and workflows, and improvements considering logging and overall reproducibility.
It also introduces two new modes of execution, allowing the user to resume workflows and re-run tasks with changed parameters without executing the complete workflow from start.
Furthermore, the tool allows detaching and reattaching from local machines without stopping jobs running on computer clusters.
The paper is written in acceptable English, the style of writing is also well readable.
The updated version of the tool provides some interesting aspects. First, the detach/reattach possibilities are a nice feature when working from laptops or desktop PCs.
Second, the inclusion of explicit tool descriptions in modules allows automated creation of a step-by-step protocol similar to a methodology draft, including citation and version information.
With other additions, the tool is adjusting to other state-of-the-art WMS, e.g. considering workflow and module sharing and the possibility of resuming interrupted workflows.
Major Comments:
Considering the reproducibility section, the comparison towards other tools is a bit biased.
For example, Watchdog 2.0 features logging of module and 3rd party software versions in a thorough manner (i.e. logging the version of all called external software).
The manuscript also mentions the use of the conda package manager in WMS as snakemake and nextflow for logging of software versions.
Yet, the topic of automated deployment of external software is mostly disregarded, even though it is one of the major advantages coming with integration of conda.
E.g. in snakemake, software versions can be fixed using either YAML files describing a conda environment or wrappers. Both ways feature not only logging but also automatic deployment of the required software version.
Therefore, reproducibility is guaranteed without the need of manual software installation.
The manuscript does not clearly talk about how and if the modules install the underlying software or if workflows automatically fetch the needed modules. While the user can just easily download all modules via the helper script, it would be tedious to manually install all external software in the correct versions.
This would be a drawback in comparision to an automatic deployment functionality, e.g. using conda.
Considering the detach/reattach functionality, the manuscript claims that snakemake terminates processes on computer clusters and waits for them to finish. In fact, when using snakemake with --cluster, jobs already running on the cluster will still keep running after the main process on the local

machine is terminated.
With the correct use of profiles, it is even possible for the workflow to check the status of those jobs after restarting the workflow.
In all fairness, this feature is pretty underdocumented in the snakemake manual.
Minor Comments:
1) When installing Watchdog via conda, javafx-sdk is installed as a dependency. Yet, when I tried to invoke the GUI, the the tool asks to set the 'JFX_SDK_LIB_PATH_ENV' variable and tries to search for javafx-sdk in ./usr and ./share, while it is located in ./lib.
While setting the path is no problem, it would be an improvement in usability if the path variable was set during installation or it the tool searched for javafx-sdk in the correct directory.
2) Similar to 1), the helper_scripts and .jar executables are hidden in the conda environment. It might make for a nicer user experience to make them callable without giving the exact path to their location. This could be done similarly to the .jar executables in GATK.
3) An online version of the Reference Book might be beneficial for users in terms of browsing the current module collection. It might be more intuitive to browse through a human readable documentation (comparable to snakemake or galaxy) than a git repository with xml files.
4) In Figure 5a, box B is in red, while other boxes are colored green. Both colors are difficult to distinguish for colorblind people (roughly 1% of women, 10% of men). I'd suggest thinking about using a red-green friendly color scheme here. This also holds for 5b to some extent.

Level of Interest

Please indicate how interesting you found the manuscript: Choose an item.

Quality of Written English

Please indicate the quality of language in the manuscript: Choose an item.

Declaration of Competing Interests

Please complete a declaration of competing interests, considering the following questions:

- Have you in the past five years received reimbursements, fees, funding, or salary from an organisation that may in any way gain or lose financially from the publication of this manuscript, either now or in the future?
- Do you hold any stocks or shares in an organisation that may in any way gain or lose financially from the publication of this manuscript, either now or in the future?
- Do you hold or are you currently applying for any patents relating to the content of the manuscript?
- Have you received reimbursements, fees, funding, or salary from an organization that holds or has applied for patents relating to the content of the manuscript?
- Do you have any other financial competing interests?

- Do you have any non-financial competing interests in relation to this paper?

If you can answer no to all of the above, write 'I declare that I have no competing interests' below. If your reply is yes to any, please give details below.

I declare that I have no competing interests

I agree to the open peer review policy of the journal. I understand that my name will be included on my report to the authors and, if the manuscript is accepted for publication, my named report including any attachments I upload will be posted on the website along with the authors' responses. I agree for my report to be made available under an Open Access Creative Commons CC-BY license (http://creativecommons.org/licenses/by/4.0/). I understand that any comments which I do not wish to be included in my named report can be included as confidential comments to the editors, which will not be published.

Choose an item.

To further support our reviewers, we have joined with Publons, where you can gain additional credit to further highlight your hard work (see: https://publons.com/journal/530/gigascience). On publication of this paper, your review will be automatically added to Publons, you can then choose whether or not to claim your Publons credit. I understand this statement.

Yes Choose an item.
