# Supplementary material for: Watchdog 2.0: New developments for reusability, reproducibility, and workflow execution
Source: Gigascience. 2020 Jun 17;9(6):giaa068. doi: 10.1093/gigascience/giaa068 (PMC7298769; doi:10.1093/gigascience/giaa068)
Supplement: giaa068_Reviewer_2_Report_Revision_1 — Jan Forster -- 5/12/2020 Reviewed [file giaa068_reviewer_2_report_revision_1.pdf]

Reviewer Report

Title: Watchdog 2.0: New developments for reusability, reproducibility and workflow execution

Version: Revision 1 **Date:** 5/12/2020

Reviewer name: Jan Forster

Reviewer Comments to Author:

The authors present their revised manuscript describing the workflow management system "Watchdog 2.0".
The manuscript seems to tackle the reviewer comments quite well, improving both the software itself and the writing and comprehensiveness of the manuscript.
Comments from my original review have been addressed satisfiedly.
An important major change in the Watchdog implementation is the possibility to automatically deploy software when using Watchdog modules.
Here, with the use of so-called execution wrappers, Watchdog is able to use predefined Conda environments and Docker images to install software. Conda environments need to be defined in the modules they refer to and can be versioned, allowing to redo analyses using different/older software environments. Execution wrappers for other package management or containerization tools are not implemented to date, but the authors claim they could be easily added using Watchdog's plugin system. This is an important step and improvement of Watchdog in terms of usability and reproducibility.
The topic of automated software deployment has also been added to the comparison with other WMS, together with the assessment of how to repurpose workflows for new datasets.
Furthermore, the authors increased overall user-friendliness by fixing path variables during installation and improving accessibility of helper scripts and similar executables.
The reference book of available Watchdog modules allows a nice and up-to-date overview for users.

Level of Interest

Please indicate how interesting you found the manuscript: Choose an item.

Quality of Written English

Please indicate the quality of language in the manuscript: Choose an item.

Declaration of Competing Interests

Please complete a declaration of competing interests, considering the following questions:

- Have you in the past five years received reimbursements, fees, funding, or salary from an organisation that may in any way gain or lose financially from the publication of this manuscript, either now or in the future?
- Do you hold any stocks or shares in an organisation that may in any way gain or lose financially from the publication of this manuscript, either now or in the future?
- Do you hold or are you currently applying for any patents relating to the content of the manuscript?
- Have you received reimbursements, fees, funding, or salary from an organization that holds or has applied for patents relating to the content of the manuscript?
- Do you have any other financial competing interests?
- Do you have any non-financial competing interests in relation to this paper?

If you can answer no to all of the above, write 'I declare that I have no competing interests' below. If your reply is yes to any, please give details below.

I declare that I have no competing interests

I agree to the open peer review policy of the journal. I understand that my name will be included on my report to the authors and, if the manuscript is accepted for publication, my named report including any attachments I upload will be posted on the website along with the authors' responses. I agree for my report to be made available under an Open Access Creative Commons CC-BY license (http://creativecommons.org/licenses/by/4.0/). I understand that any comments which I do not wish to be included in my named report can be included as confidential comments to the editors, which will not be published.

Choose an item.

To further support our reviewers, we have joined with Publons, where you can gain additional credit to further highlight your hard work (see: https://publons.com/journal/530/gigascience). On publication of this paper, your review will be automatically added to Publons, you can then choose whether or not to claim your Publons credit. I understand this statement.

Yes Choose an item.
