# Supplementary material for: Watchdog 2.0: New developments for reusability, reproducibility, and workflow execution
Source: Gigascience. 2020 Jun 17;9(6):giaa068. doi: 10.1093/gigascience/giaa068 (PMC7298769; doi:10.1093/gigascience/giaa068)
Supplement: giaa068_Reviewer_3_Report_Original_Submission — Stian Soiland-Reyes -- 2/13/2020 Reviewed [file giaa068_reviewer_3_report_original_submission.pdf]

Reviewer Report

Title: Watchdog 2.0: New developments for reusability, reproducibility and workflow execution

Version: Original Submission **Date:** 2/13/2020

Reviewer name: Stian Soiland-Reyes

Reviewer Comments to Author:

Hi, I am Stian Soiland-Reyes https://orcid.org/0000-0001-9842-9718 and have pledged the Open Peer Review Oath https://doi.org/10.12688/f1000research.5686.2:

Principle 1: I will sign my name to my review

Principle 2: I will review with integrity

Principle 3: I will treat the review as a discourse with you; in particular, I will provide constructive criticism

Principle 4: I will be an ambassador for the practice of open science

This review is licensed under a Creative Commons Attribution 4.0 International License http://creativecommons.org/licenses/by/4.0/ and is also available at the (For now) secret URL https://gist.github.com/stain/3405503f207c703efc48e8d145dd3bb6

This paper presents a workflow system Watchdog, and in particular improvements made since its earlier release.

As a developer and user of multiple workflow systems I found this article inspiring, as it shows that a workflow system can combine usability aspects such as tool documentation and drag-and-drop design with more advanced workflow engine features such as detach/resume.

The developers of WatchDog have taken on board features and practices emerging in other workflow system, for instance the establishment of a community workflow repository similar to Nextflow's nf-core, or the generation of software citations and reports on individual tools used.

Notably in this software is the lack of use containers (Docker, Singularity) or software package systems (Conda, Debian-Med) to reliably execute bioinformatics tools. The authors have however made Watchdog itself available as both a Docker container and BioConda package. Watchdog do helpfully include a script to check if tool dependencies are installed, but do not seem to be able to help with installing missing tools like bowtie. While full-blown container support might require significant developer effort, a simpler approach like Galaxy and Snakemake's support for Conda environments would be useful and improve reproducibility, particularly when executing Watchdog workflows on more than one computer system.

Beyond a tickbox comparison, the case is not well made for why bioinformatics need another workflow system, particularly as no reflection is done on making a custom workflow language as opposed to basing it (or importing/exporting) an standard-based approaches like WDL or CWL. Nevertheless I think this article is welcome, as I believe the more mainstream workflow system developers have several lessons to be learnt from Watchdog.

--

Note - the below questions from GigaScience guidelines mainly relate to *data*, which I here interpret as *software* as no data is relevant to this Technical Note.

> Q1: Is the rationale for collecting and analyzing the data well defined?

This work is about software, which has been provided, but it is not large-scale. Support for distributed computing (SSH, Slurm) is mentioned, but no further consideration cloud computing is highlighted.

The software is well described, including references to an earlier article on the previous version.

> Q2: Is it clear how data was collected and curated?

No particular data was collected, but attribution is given for software creation.

> Q3: Is it clear - and was a statement provided - on how data and analyses tools used in the study can be accessed?

GitHub links are provided, as well as documentation pages.

> Q4: Are accession numbers given or links provided for data that, as a standard, should be submitted to a community approved public repository?

GitHub URLs are provided. Documentation is hosted on their institutional website.

For longevity beyond GitHub, I would have preferred to also see DOIs for Zenodo-GitHub-synced releases, see https://guides.github.com/activities/citable-code/

As for the https://github.com/watchdog-wms/ community repositories they may not have regular "versions" as such (each module have their internal version number), but should have timestamped releases/tags and equivalent Zenodo DOIs.

Similarly I would have preferred to see a snapshot of the documentation page(s) in Zenodo or http://web.archive.org/ to guard against changes in institutional infrastructure - in particular the use of https://rawgit.com/ for documentation should be avoided as it has been decommissioned.

> Q5: Is the data and software available in the public domain under a Creative Commons license?

The source code of the workflow system, community workflows and modules are all licensed under the OSI-approved GNU GPL v3.

I have raised two issues in this regard:

Users should be made aware of the license of the community workflows in case they want to copy and modify them https://github.com/watchdog-wms/watchdog-wms-workflows/issues/13

Users should be aware of license of the community modules and the implication of using GPLv3-licensed modules from their workflows (alternatively a different license should be considered for modules) https://github.com/watchdog-wms/watchdog-wms-modules/issues/89

> Q6: Are the data sound and well controlled?

The software source code is well organized. No particular biological claims are made in this Technical Report.

The license of open source dependencies is not propagated in the software distribution, I raised this as https://github.com/klugem/watchdog/issues/4

> Q7: Is the interpretation (Analysis and Discussion) well balanced and supported by the data?

The interpretation should discuss the relevance of all the results in an unbiased manner. Are the interpretations overly positive or negative? Note that the authors may include opinions and speculations in an optional 'Potential Implications' section of the manuscript; thus, if there is material in other parts of the manuscript that you feel would be better suited in such a section, please state that. Conclusions drawn from the study should be valid and result directly from the data shown, with reference to other

relevant work as applicable. Have the authors provided references wherever necessary?
The article is understandably biased in favour of the described software, which should be modulated. For instance, table 1 compares features with other workflow system, but the features seem arbitrarily selected to favour the author's system. For instance I would add estimate of number of bioinformatics tools available, if commercial support is available, if there's a web interface, data visualization, if inputs/outputs are implicit or explit; scattering and parallelization support, and (if table space permitted) list of supported computation backends.
The abstract boldly claims the software "ensures reproducibility of results", but the community workflows are not re-usable without cumbersome installation of their dependent bioinformatics tools, and modifications of absolute file paths like /usr/local/storage in the module and workflow definitions. Some support for parameterization and Docker containers is possible in WatchDog, which should improve on these reproducibility concerns, but in practice this is not done, and the manuscript should reflect this as a current limitation and future aim.
> Q8: Are the methods appropriate, well described, and include sufficient details and supporting information to allow others to evaluate and replicate the work?
After request from the reviewer https://github.com/klugem/watchdog/issues/3 the developers responded quickly to add a reproducible build script based on Apache Maven.
I have not assessed the suitability or correctness of the bioinformatics community modules in https://github.com/watchdog-wms/watchdog-wms-modules or workflows in https://github.com/watchdog-wms/watchdog-wms-workflows - but observe that they include the use of familiar tools like bowtie2, samtools and fastQC.
> Q9: What are the strengths and weaknesses of the methods?
This paper proposes WatchDog 2.0 as a workflow system to be used for bioinformatics pipelines. There are quite a few existing workflow system, https://s.apache.org/existing-workflow-systems currently list more than 270, . The paper includes a comparison to well-known systems Galaxy, Nextflow, Snakemake and KNIME. The Common Workflow Language standard https://www.commonwl.org/ and its multiple implementations are however not mentioned, although that effort was started primarily as a reaction to the growing plethora of bioinformatics workflow systems. (full disclosure: this reviewer is on the CWL Leadership Team)
The design of this workflow system combines many strengths of the more mainstream workflow systems with a strong focus on tool documentation. It is a common problem for bioinformatics pipelines, particularly in collaborative design, to document and understand the different parameters, and I particularly like the generation of module documentation and the module maker tool for generating tool definitions based on --help output.
It is interesting that this workflow system uses XML Schema as the basis for tool definitions, and that gives a solid and validateable basis for their use in workflow definitions. However XML do unfortunately represent a barrier of entry as many bioinformaticians now are less familiar with XML and are expecting text formats like JSON and YAML, or a script-like workflow domain-specific languages (DSL) as in Nextflow and Snakemake. The manuscript should highlight this as a concern, and assure the reader that the barrier is lowered by the GUIs provided in workflow designer and module maker. Examples should be given for recommended XML editors.
Bioinformatics workflows are naturally data/file-driven. What could perhaps be unusual to some

newcomers to Watchdog is the reliance on absolute paths and indirect passing of values by filenames hidden inside tool definitions, and the fact that users have to both provide a data dependency (by having the outputted filename given as a filename to the next step) and a control dependency (to ensure the next step is not executed too early). Most workflow systems figure out task dependencies based on explicitly connected inputs and outputs, allow parameterization so a workflow can be repurposed without editing it for every run, in Watchdog one has to inspect the tool XSD to know which output files it might create and thus implicitly connect.
In many sense WatchDog is here following the same approach as Snakemake's file patterns, this is particularly shown in examples that involve iterations where the following steps need to embed the iteration number macro as part of their input declaration. These macros-in-XML e.g. {%EXAMPLE_DATA%} are powerful, but also another barrier to entry for users, and not currently well-handled in the GUI nor explained well enough in the user manual. In a sense writing WatchDog workflow therefore involve 4 languages or DSLs that the user to a needs to learn: 1) XML Schema with extension for defining modules; 2) WatchDog XML for defining the workflow; 3) macros for defining the file names and parameters; and 4) shell script wrappers that unpack the arguments to an actual command line
The user manual is quite focused on the XML, and probably needs to be updated to reflect the improved GUI of WatchDog 2.0, as many of the same considerations are still relevant to the GUI use, e.g. the use of macros and absolute file names as a way to pass data.
> Q10: Have the authors followed best-practices in reporting standards?
This technical report describes another workflow system that is similar in functionality to Taverna and Galaxy and provides similar reproducibility aspects for its users.
> Q11: Can the writing, organization, tables and figures be improved?
The language, organization and figures are of a good quality. However the arguments seems to be targeting software engineers rather than the typical Gigascience bioinformatics users. It might be relevant to briefly describe what kind of bioinformatics workflows the Watchdog community has already developed, and which bioinformatics tools are currently supported.
> Q12: When revisions are requested.
I have requested some minor revisions as detailed in the rest of this review
1. Zenodo/FigShare DOI for code release
2. Modulate wf system comparison to be more neutral
3. Admit usability concerns in terms of reliance on XML, hard-coded paths and macro language
4. Address GPLv3 licensing questions for the community repositories
5. Admit current lack of container/conda support and thus reproducibility/portability issue
6. Adjust language to reflect bioinformatics audience
> Q13: Are there any ethical or competing interests issues you would like to raise?
No ethical issues or competing interests have been declared by the authors nor identified by the reviewer.

Level of Interest

Please indicate how interesting you found the manuscript: Choose an item.

Quality of Written English

Please indicate the quality of language in the manuscript: Choose an item.

Declaration of Competing Interests

Please complete a declaration of competing interests, considering the following questions:

- Have you in the past five years received reimbursements, fees, funding, or salary from an organisation that may in any way gain or lose financially from the publication of this manuscript, either now or in the future?
- Do you hold any stocks or shares in an organisation that may in any way gain or lose financially from the publication of this manuscript, either now or in the future?
- Do you hold or are you currently applying for any patents relating to the content of the manuscript?
- Have you received reimbursements, fees, funding, or salary from an organization that holds or has applied for patents relating to the content of the manuscript?
- Do you have any other financial competing interests?
- Do you have any non-financial competing interests in relation to this paper?

If you can answer no to all of the above, write 'I declare that I have no competing interests' below. If your reply is yes to any, please give details below.

I declare that I have no competing interests. In my review I refer to Common Workflow Language, of which I am on the Leadership Team.

I agree to the open peer review policy of the journal. I understand that my name will be included on my report to the authors and, if the manuscript is accepted for publication, my named report including any attachments I upload will be posted on the website along with the authors' responses. I agree for my report to be made available under an Open Access Creative Commons CC-BY license (http://creativecommons.org/licenses/by/4.0/). I understand that any comments which I do not wish to be included in my named report can be included as confidential comments to the editors, which will not be published.

Choose an item.

To further support our reviewers, we have joined with Publons, where you can gain additional credit to further highlight your hard work (see: https://publons.com/journal/530/gigascience). On publication of this paper, your review will be automatically added to Publons, you can then choose whether or not to claim your Publons credit. I understand this statement.

Yes Choose an item.
