# Supplementary material for: Watchdog 2.0: New developments for reusability, reproducibility, and workflow execution
Source: Gigascience. 2020 Jun 17;9(6):giaa068. doi: 10.1093/gigascience/giaa068 (PMC7298769; doi:10.1093/gigascience/giaa068)
Supplement: giaa068_Reviewer_3_Report_Revision_1 — Stian Soiland-Reyes -- 5/19/2020 Reviewed [file giaa068_reviewer_3_report_revision_1.pdf]

Reviewer Report

Title: Watchdog 2.0: New developments for reusability, reproducibility and workflow execution

Version: Revision 1 **Date:** 5/19/2020

Reviewer name: Stian Soiland-Reyes

Reviewer Comments to Author:

The authors have responded well to the reviewer comments, and made several improvements to the software and its documentation. It is particularly noteworthy that after encouragement from reviewers WatchDog developers have now added full Conda support for modules.

I do not understand from the manuscript, examples or the code documentation how to use the Docker execution runtime, as it necessarily would need to specify the correct container for different modules, but unlike the Conda environments, these seem not to be provided in the included modules, except in the RNA_DifferentialGeneExpression where this is shown by using the conda/miniconda3 Docker container (and then software installed using conda inside), which I guess is an acceptable, if not ideal workaround compared to having separate containers for each task.

I remain concerned about the complexity for users to learn the different languages and syntaxes, however the publication of module handbooks and addition of the macro documentation allow this to happen in a gradual manner. The user interface of workflow designer in WatchDog 2.0 still remains fairly technical as it is closer to the XML being edited rather than the users' view of tool composition - e.g. the user has to cross-check against the module handbooks (or XSD) to find the input filenames that needs to be connected from previous step's output; the outputs are not presented in the UI. I tried to point out this in my previous review, but may have muddled my language.

The combination of having to do "run-after" coordination (which I agree is *sometimes* useful) and manual data dependency can be a source of bugs in workflow authoring - e.g. a user could ask for step A to run before step B, while step B needs an output from step A. I have not tested if the engine can check for such inconsistency before running, or if it will accidentally use old file data.

Following reviewer comments, the releases of the software has been greatly improved, with DOIs for code citations, improved build system and more liberal licensing for the workflow modules.

The improvements to the manuscript address most of the previous concerns raised by the reviewers and help clarify the new Conda features. The language is still a bit "sales brochure" like and could do with some neutral tones.

Level of Interest

Please indicate how interesting you found the manuscript: Choose an item.

Quality of Written English

Please indicate the quality of language in the manuscript: Choose an item.

Declaration of Competing Interests

Please complete a declaration of competing interests, considering the following questions:

- Have you in the past five years received reimbursements, fees, funding, or salary from an organisation that may in any way gain or lose financially from the publication of this manuscript, either now or in the future?
- Do you hold any stocks or shares in an organisation that may in any way gain or lose financially from the publication of this manuscript, either now or in the future?
- Do you hold or are you currently applying for any patents relating to the content of the manuscript?
- Have you received reimbursements, fees, funding, or salary from an organization that holds or has applied for patents relating to the content of the manuscript?
- Do you have any other financial competing interests?
- Do you have any non-financial competing interests in relation to this paper?

If you can answer no to all of the above, write 'I declare that I have no competing interests' below. If your reply is yes to any, please give details below.

Non-financial competing interest as a member of the Common Workflow Language (CWL) Leadership team.

I agree to the open peer review policy of the journal. I understand that my name will be included on my report to the authors and, if the manuscript is accepted for publication, my named report including any attachments I upload will be posted on the website along with the authors' responses. I agree for my report to be made available under an Open Access Creative Commons CC-BY license (http://creativecommons.org/licenses/by/4.0/). I understand that any comments which I do not wish to be included in my named report can be included as confidential comments to the editors, which will not be published.

Choose an item.

To further support our reviewers, we have joined with Publons, where you can gain additional credit to further highlight your hard work (see: https://publons.com/journal/530/gigascience). On publication of this paper, your review will be automatically added to Publons, you can then choose whether or not to claim your Publons credit. I understand this statement.

Yes Choose an item.
